# π-π Conjugation Enhances Oligostilbene’s Antioxidant Capacity: Evidence from α-Viniferin and Caraphenol A

**DOI:** 10.3390/molecules23030694

**Published:** 2018-03-19

**Authors:** Xican Li, Yulu Xie, Hong Xie, Jian Yang, Dongfeng Chen

**Affiliations:** 1 School of Chinese Herbal Medicine, Guangzhou University of Chinese Medicine, Waihuan East Road No. 232, Guangzhou Higher Education Mega Center, Guangzhou 510006, China; xieyulu1900@163.com (Y.X.); xiehongxh1@163.com (H.X.); javons@yeah.net (J.Y.); 2 Innovative Research & Development Laboratory of TCM, Guangzhou University of Chinese Medicine, Waihuan East Road No. 232, Guangzhou Higher Education Mega Center, Guangzhou 510006, China; 3 School of Basic Medical Science, Guangzhou University of Chinese Medicine, Guangzhou 510006, China; 4 The Research Center of Basic Integrative Medicine, Guangzhou University of Chinese Medicine, Waihuan East Road No. 232, Guangzhou Higher Education Mega Center, Guangzhou 510006, China

**Keywords:** oligostilbene, stilbene trimer, α-viniferin, caraphenol A, antioxidant, conjugative double bond

## Abstract

α-Viniferin and caraphenol A, the two oligostilbenes, have the sole difference of the presence or absence of an exocyclic double bond at the π-π conjugative site. In this study, the antioxidant capacity and relevant mechanisms for α-viniferin and caraphenol A were comparatively explored using spectrophotometry, UV-visible spectral analysis, and electrospray ionization quadrupole time-of-flight tandem mass spectrometry (UPLC–ESI–Q–TOF–MS/MS) analysis. The spectrophotometric results suggested that caraphenol A always gave lower IC_50_ values than α-viniferin in cupric ion-reducing antioxidant capacity assay, ferric-reducing antioxidant power assay, 1,1-diphenyl-2-picryl-hydrazl radical (DPPH•)-scavenging, and 2-phenyl-4,4,5,5-tetramethylimidazoline-1-oxyl 3-oxide radical-scavenging assays. In UV-visible spectra analysis, caraphenol A was observed to show enhanced peaks at 250–350 nm when mixed with Fe^2+^, but α-viniferin exhibited no similar effects. UPLC–ESI–Q–TOF–MS/MS analysis revealed that α-viniferin mixed with DPPH• produced radical adduct formation (RAF) peak (*m/z* = 1070–1072). We conclude that the antioxidant action of α-viniferin and caraphenol A may involve both redox-mediated mechanisms (especially electron transfer and H^+^-transfer) and non-redox-mediated mechanisms (including Fe^2+^-chelating or RAF). The π-π conjugation of the exocyclic double bond in caraphenol A can greatly enhance the redox-mediated antioxidant mechanisms and partially promote the Fe^2+^-chelating mechanism. This makes caraphenol A far superior to α-viniferin in total antioxidant levels.

## 1. Introduction

**Figure 1:**
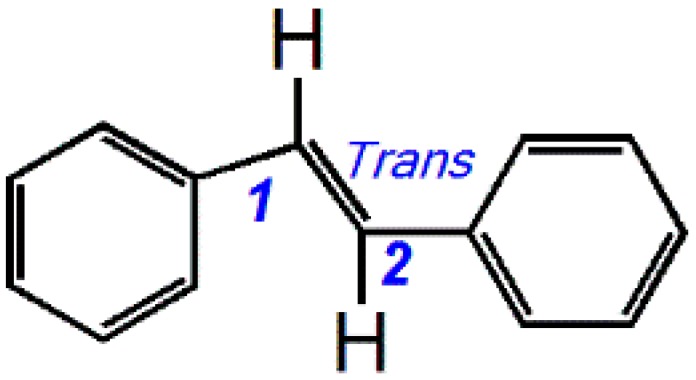
The *trans*-1,2-diphenylethene core.

In natural product chemistry, the term “stilbene” refers to the *trans*-1,2-diphenylethene core (Figure 1). The 1,2-double bond (C=C) can induce polymerization to form oligostilbenes, mainly dimeric stilbene, trimeric stilbene, and tetrameric stilbene. To date, at least 200 natural oligostilbenes have been discovered [1,2]. Almost all of these natural oligostilbenes are documented to bear phenolic -OH [2], and thus they can be regarded as the members of natural phenolics and function as phenolic antioxidants [3].

**Figure 2:**
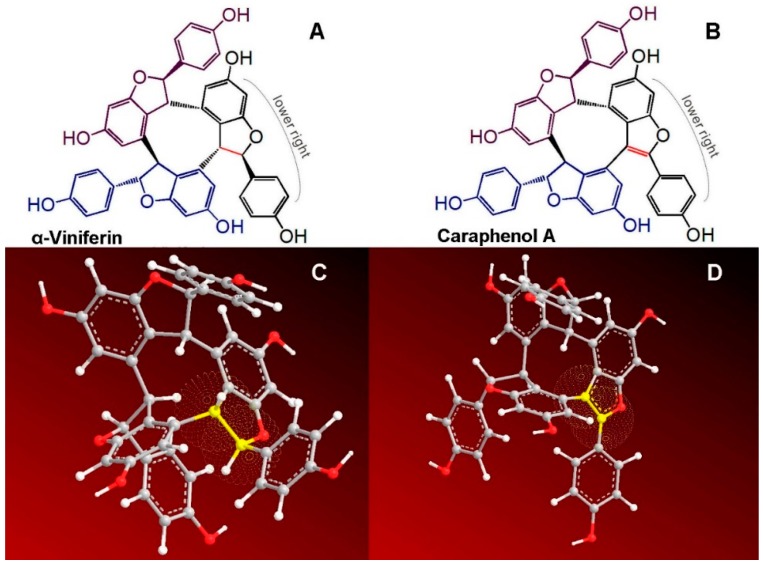
Structures and preferential conformation-based ball-stick models of reference compounds: (**A**) the structure of α-viniferin; (**B**) the structure of caraphenol A; (**C**) the preferential conformation-based ball-stick model of α-viniferin; (**D**) the preferential conformation-based ball-stick model of caraphenol A. (The ball-stick models were created in Chem3D Pro 14.0).

Recently, three dimers of resveratrol have been found in grape wine, including parthenocissin A, quadrangularin A, and pallidol [4]. They could effectively scavenge DPPH• radical and singlet oxygen (^1^O_2_). In addition, two trimeric stilbenes, α-viniferin and caraphenol A (Figure 2), have been isolated from *Caragana sinica* [5,6]. α-Viniferin was suggested to have an inhibitory effect on lipid peroxidation, while caraphenol A was observed to inhibit human immunodeficiency virus (HIV) [6]. Nevertheless, there has been no antioxidant structure-activity relationship study of oligostilbenes until now, to the best of our knowledge.

Structurally, oligostilbenes are quite different from other natural phenolics, such as flavonoids, phenolic acids, and phenolic alkaloids [7]. As seen in Figure 2, caraphenol A has an exocyclic double bond linking the lower right phenyl ring to the lower right benzofuran-fused-ring, while at the corresponding site, α-viniferin has an exocyclic single bond rather than a double bond (Figure 2A). Undoubtedly, the sole difference between the two trimeric stilbenes is attributed to the bond type (single or double bond). However, according to fundamental organic chemistry, while the exocyclic double bond in caraphenol A can conjugate the phenyl ring with the benzo[*b*]furan-fused-ring, the single bond in α-viniferin cannot conjugatively link the phenyl ring with the benzo[*b*]furan-fused-ring. Thus, the essential difference between the two trimeric stilbenes is the presence or absence of π-π conjugation between the lower right phenyl ring and the lower right benzo[*b*]furan-fused-ring (Figure 2A,B). Thereby, α-viniferin and caraphenol A can act as an ideal pair of oligostilbenes to explore the possible effect of π-π conjugation towards the antioxidant capacity of oligostilbenes.

In this study, we attempted to comparatively determine their antioxidant capacities using spectrophotometry, UV-vis spectral analysis, and electrospray ionization quadrupole time-of-flight tandem mass spectrometry (UPLC–ESI–Q–TOF–MS/MS) analysis. These determinations will provide evidence regarding the effect of π-π conjugation towards antioxidant oligostilbenes (natural phenolics).

## 2. Results and Discussion

It is reported that, under atmospheric conditions or during cellular metabolism, oxygen (O_2_) can be transformed into various reactive oxygen species (ROS), such as •OH radicals and •O_2_^−^ radicals [8]. Since excessive ROS are harmful to cells [9], animals and plants find ways to scavenge them [8,10]. This ROS-scavenging action is termed as antioxidation in free radical biology and medicine. Accumulative evidence suggests that antioxidation is a complicated process involved in several pathways. Put simply, these pathways can be divided into redox-mediated pathways and non-redox-mediated pathways [11].

Redox-mediated pathways are characterized by electron transfer (ET), through which electrons are transferred from a (phenolic) antioxidant to ROS or reactive nitrogen species (RNS) [12]. In order to test the ET possibility of α-viniferin and caraphenol A, these two trimeric stilbenes were analyzed using a ferric-reducing antioxidant power (FRAP) assay. The FRAP assay is a colorimetric analysis performed at a pH of 3.6 [13]. This low pH value is thought to suppress H^+^ ionization, and thus, the FRAP is proposed to be a mere ET process [14]. As shown in Supplementary 1, the FRAP percentages of caraphenol A increased with concentration, while those of α-viniferin hardly increased with concentration. This clearly suggested that caraphenol A had much higher ET potential than α-viniferin at a lower, acidic pH.

In order to explore whether the two trimeric stilbenes transfer electrons at physiological pH, we also conducted a cupric ion-reducing antioxidant capacity (CUPRAC) assay. The CUPRAC assay has been demonstrated to be an ET-based Cu^2+^-reducing reaction in pH 7.4 solution [15]. As illustrated in Supplementary 1, the two trimeric stilbenes reduced Cu^2+^ to Cu^+^ in good agreement with the dosage. This suggests an ET potential of the two trimeric stilbenes at physiological pH.

The above FRAP and CUPRAC assays actually involved the interaction of trimeric stilbenes with metal ions. There was no direct interaction of trimeric stilbenes with free radicals. In order to observe the direct interaction of the two trimeric stilbenes with free radicals, each trimeric stilbenes was mixed with 1,1-diphenyl-2-picryl-hydrazyl radical (DPPH•) in a methanol solution. As seen in Supplementary 1, α-viniferin and caraphenol A could increase the DPPH•-scavenging percentages depending on the dosage. DPPH•-scavenging, however, is proven to involve in ET and H^+^-transfer pathways [16,17,18,19,20,21]. These pathways are essentially mediated by redox reactions, thus indicating that α-viniferin and caraphenol A may undergo redox reactions (especially ET and H^+^-transfer) to scavenge DPPH•.

In order to study the radical-scavenging action of α-viniferin and caraphenol A further, they were measured using a PTIO•-scavenging assay, a method newly established by our research group [22]. In the PTIO•-scavenging assay, α-viniferin and caraphenol A could also increase the scavenging percentages depending on their concentrations (Supplementary 1). Similar to DPPH•-scavenging, PTIO•-scavenging is also involved in ET and H^+^-transfer [22,23]. This further supported the assumption from the above DPPH•-scavenging assay.

The IC_50_ value in μM was obtained from the dose-response curves in Supplementary 1. The IC_50_ values with different letters (a, b, or c) in the same assay are significantly (*p* < 0.05) different among α-viniferin, caraphenol A, and the positive control Trolox.

**Figure 3:**
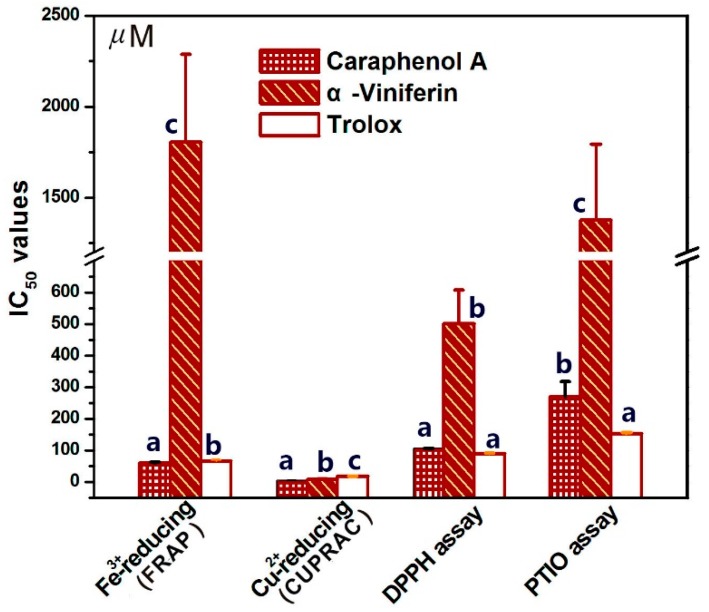
The IC_50_ values of α-viniferin and caraphenol A in antioxidant assays, including the ferric-reducing antioxidant power (FRAP) assay, cupric ion-reducing antioxidant capacity (CUPRAC) assay, DPPH•-scavenging assay, and PITO•-scavenging assay.

To quantitatively evaluate their antioxidant levels in the FRAP assay, CUPRAC assay, DPPH•-scavenging assay, and PITO•-scavenging assay, their IC_50_ values were calculated and are shown in Figure 3. According to the IC_50_ values in Figure 3, the ratio value of IC_50,α-viniferin_:IC_50,caraphenol A_ in the FRAP assay was calculated as 29.9. Meanwhile, the ratio values in the CUPRAC assay, DPPH•-scavenging assay, and PITO•-scavenging assay were 1.7, 4.7, and 5.1, respectively. The average ratio value was obtained as 10.4. This means that the total antioxidant capacity of caraphenol A is about 10.4-fold higher than that possessed by α-viniferin.

This difference can be attributed only to the exocyclic carbon-carbon bond type (C-C or C=C). The exocyclic double bond in caraphenol A conjugates the lower right phenyl ring and the lower right benzofuran-fused-ring in Figure 2B, thus considerably extending the molecular conjugative system. Density functional response theory indicated that the extended π-π conjugation had a stronger capability to stabilize the radical species via delocalization of the π-electrons [24]. Thereby, π-π conjugation in caraphenol A greatly enhances redox-mediated antioxidant capacity. This can explain the aforementioned great difference between α-viniferin and caraphenol A in the FRAP, DPPH•-scavenging, and PITO•-scavenging assays (Figure 3), and the previous findings that *trans*-*ε*-viniferin (IC_50_ 62.5 ± 0.8 μM) possessed a higher DPPH•-scavenging level than (+)-ampelopsin A (IC_50_ > 200 μM) [25].

**Figure 4:**
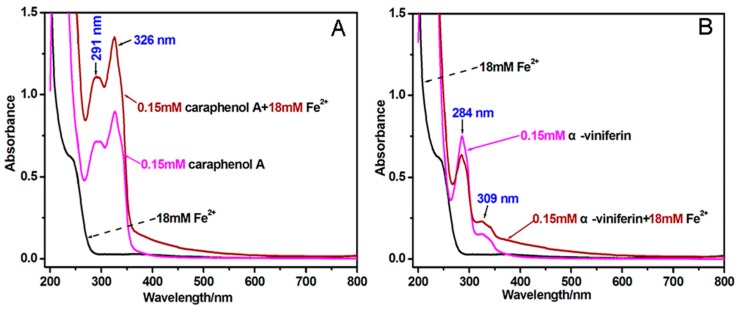
UV spectra of Fe^2+^-chelating with caraphenol A (**A**) and α-viniferin (**B**).

A wide range of studies have pointed out that, apart from redox-mediated pathways, non-redox pathways may also occur during antioxidant processes [26,27,28,29]. That the non-redox pathways may include transition metal chelating can be justified by the fact that transition metals (especially Fe^2+^) function as catalysts to accelerate the generation of ROS. For instance, Fe^2+^ can catalyze H_2_O_2_ to produce •OH radical via the Fenton reaction. Fe^2+^-chelation can thus block •OH radical production. In the study, we used UV-vis spectra to monitor the Fe^2+^-chelation reactions of α-viniferin and caraphenol A. As seen in Figure 4, caraphenol A with π-π conjugation gave stronger UV-visible peaks at 250–350 nm when mixed with Fe^2+^. α-Viniferin showed no similar changes after mixing with Fe^2+^. This indicates that π-π conjugation promotes the Fe^2+^-chelation capacity of phenolic oligostilbenes to some extent.

**Figure 5:**
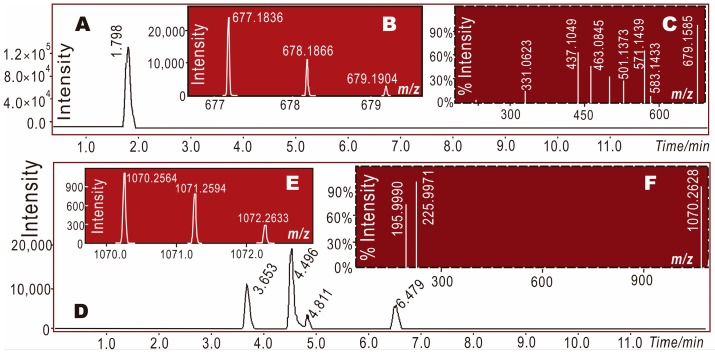
The main results of UPLC–ESI–Q–TOF–MS/MS analysis: (**A**) Total ion chromatographical diagram of α-viniferin; (**B**) Primary MS spectra (i.e., molecular ion peaks) of α-viniferin; (**C**) Secondary MS spectra (i.e., the fragment peaks) of α-viniferin; (**D**) Total ion chromatographical diagram of α-viniferin-DPPH• extracted by C_60_H_41_N_5_O_15_; (**E**) Primary MS spectra (i.e., molecular ion peaks) of α-viniferin-DPPH•; (**F**) Secondary MS spectra (i.e., the fragment peaks) of α-viniferin-DPPH•.

It is worth mentioning that a radical adduct formation (RAF) product was also found in the mixture of α-viniferin with DPPH• radical. Through the UPLC–ESI–Q–TOF–MS/MS analysis, a molecular ion peak was obtained at an *m*/*z* value of 1070–1072 (Figure 5E), i.e., an RAF product of α-viniferin-DPPH was obtained. RAF occurs only after hydrogen atom transfer (HAT) or the deprotonation of an antioxidant molecule [7,28,30,31], and thus can be considered as the decay product of an antioxidant radical [32]. However, some studies have also suggested it as a minor non-redox-mediated antioxidant pathway [33].

There were four peaks in the total ion chromatographical diagram of α-viniferin-DPPH• in Figure 5D, i.e., retention times = 3.653, 4.496, 4.811, and 6.479 min. Each of the four peaks exhibited a primary MS spectrum, as shown in Figure 5E. The secondary MS spectra of the latter three peaks are shown in Figure 5F, while the first peak was too small to give rise to a secondary MS spectrum. Their original spectra are detailed in Supplementary 2.

## 3. Materials and Methods

### 3.1. Chemicals 

α-Viniferin (CAS 62218-13-7, C_42_H_30_O_9_, M.W. 678.7, purity 97%, yellow powder, Supplementary 3) and caraphenol A (CAS 354553-35-8, C_42_H_28_O_9_, M.W. 676.7, purity 97%, brown powder, Supplementary 3) were obtained from BioBioPha Co., Ltd. (Kunming, China). The 2-phenyl-4,4,5,5-tetramethylimidazoline-1-oxyl-3-oxide radical (PTIO•) was from TCI Chemical Co. (Shanghai, China). The 1,1-diphenyl-2-picryl-hydrazl radical (DPPH•), (±)-6-hydroxyl-2,5,7,8-tetramethlychromane-2-carboxylic acid (Trolox), 2,4,6-tripyridyltriazine (TPTZ), and 2,9-dimethyl-1,10-phenanthroline (neocuproine) were purchased from Sigma-Aldrich Shanghai Trading Co. (Shanghai, China). Ultrapure water was obtained using a Milli-Q system (Millipore, Bedford, NY, USA). Methanol was of HPLC grade. FeCl_2_·4H_2_O (A.R.), FeCl_3_·6H_2_O (A.R.), CH_3_COOH (A.R.), CH_3_COONH_4_ (A.R.), CuSO_4_ (A.R.), KH_2_PO_4_ (A.R.), and Na_2_HPO_4_·12H_2_O (A.R.) were from Guangdong Guanghua Chemical Plants Co., Ltd. (Shantou, China).

### 3.2. FRAP Assay (Fe^3+^-Reducing Assay)

The Fe^3+^-reducing assay was established by Benzie and Strain and is formally named as FRAP [15]. The experimental protocol of this assay was described in a previous report [34]. Briefly, the FRAP reagent was freshly prepared by mixing 10 mM TPTZ, 20 mM FeCl_3_, and 0.25 M acetate buffer at a ratio of 1:1:10 at pH 3.6. The test sample (*x* = 4–20 μL, 0.2 mg/mL) was added to (20 − *x*) μL of 95% ethanol followed by 80 μL of FRAP reagent. After a 30-min incubation at ambient temperature, the absorbance was measured at 595 nm using a microplate reader (Multiskan FC, Thermo Scientific, Shanghai, China). The relative reducing power of the sample was calculated using the following formula:

$$\mathrm{Relative}\;\mathrm{reducing}\;\mathrm{effect}\;\%\;=\frac{A-A_{min}}{A_{max}-A_{min}}\times 100\%$$

where *A_max_* was assigned as 1.41, and *A_min_* is the minimum absorbance in the test. *A* is the absorbance of the sample.

### 3.3. CUPRAC Assay (Cu^2+^-Reducing Assay)

This assay was carried out according to the method described by Wang [35]. Briefly, 12 μL of CuSO_4_ aqueous solution (10 mmol/L), 12 μL of neocuproine ethanolic solution (7.5 mmol/L), and (75 − x) μL of ammonium acetate buffer solution (0.1 mol/L, pH 7.5) were added to wells with different volumes of sample (0.05 mg/mL, 4–20 μL). The absorbance at 450 nm after 30 min was measured using the aforementioned microplate reader. The relative CUPRAC power was calculated using the formula for FRAP. *A_max_* was assigned as 0.159.

### 3.4. PTIO•-Scavenging Assay

The PTIO•-scavenging assays were conducted based on our method [7]. In brief, the test sample solution (*x* = 0–20 μL, 1 mg/mL) was added to (20 − *x*) μL of 95% ethanol, followed by 80 μL of an aqueous PTIO• solution. The aqueous PTIO• solution was prepared using a phosphate-buffer solution (0.1 mM, pH 7.0). The mixture was maintained at 37 °C for 2 h, and the absorbance was then measured at 560 nm using the aforementioned microplate reader. The PTIO• inhibition percentage was calculated as follows:

$$\mathrm{Inhibition}\%=\frac{A_{0}-A}{A_{0}}\times 100\%$$

where A_0_ is the absorbance of the control without the sample, and A is the absorbance of the reaction mixture with the sample.

### 3.5. DPPH•-Scavenging Assay

DPPH• radical-scavenging activity was determined as described previously [36]. Briefly, 80 μL DPPH•-methanolic solution (0.1 mol/L) was mixed with 0.5 mg/mL sample-methanolic solution (4–20 μL). The mixture was maintained at room temperature for 30 min, and the absorbance was measured at 519 nm on a microplate reader. The percentage of DPPH• scavenging activity was calculated based on the formula presented in Section 3.4.

### 3.6. Determining Fe^2+^-Chelating Ability of α-Viniferin and Caraphenol A (UV-Visible Spectra Analysis)

The method was based on the previous study [37]. Briefly, 500 μL of a methanolic solution of α-viniferin (0.15 mmol/L) was added to 500 μL of an aqueous solution of FeCl_2_•4H_2_O (18 mmol/L). The solution was then mixed vigorously. Then, the mixture was kept for 30 min at room temperature and the spectrum was obtained using a UV-visible spectrophotometer (Unico UV 2600A, Shanghai, China) from 200–800 nm.

### 3.7. Electrospray Ionization Quadrupole Time-of-Flight Tandem Mass Spectrometry (UPLC–ESI–Q–TOF–MS/MS) Determining DPPH• Reaction Products with α-Viniferin or Caraphenol A 

The DPPH• reaction products with α-viniferin (or caraphenol A) were prepared based on a previous work [17]. In brief, methanolic solution of α-viniferin (or caraphenol A) was mixed with a solution of DPPH• radical in methanol at a molar ratio of 1:2, and the mixture was incubated for 24 h at room temperature. The product was then filtered through a 0.22-μm filter for UPLC-ESI-Q-TOF-MS/MS analysis [22].

In the UPLC analysis, the sample solution (1 μL) was injected to a C_18_ column (2.0 mm × 100 mm, 1.6 μm, Phenomenex Co., Torrance, USA). The mobile phase, used for the elution of the system, consisted of a mixture of methanol (phase A) and ultrapure water (phase B). The products mixture was eluted at a flow rate of 0.3 mL/min with the following gradient elution program: 0–10 min, 60–100% A; 10–15 min, 100% A. The column temperature was 40 °C. ESI-Q-TOF-MS/MS analysis was performed on a Triple TOF 5600^plus^ Mass spectrometer (AB SCIEX, Framingham, MA, USA) equipped with an ESI source, which was run in the negative ionization mode. The system was run with the following parameters: ion spray voltage, −4500 V; ion source heater, 550 °C; curtain gas (CUR, N_2_), 30 psi; nebulizing gas (GS1, Air), 50 psi; Tis gas (GS2, Air), 50 psi. The declustering potential (DP) was set at −100 V, whereas the collision energy (CE) was set at −40 V with a collision energy spread (CES) of 20 V. The RAF MS peaks were extracted using the corresponding formula (e.g., [C_42_H_30_O_9_ − H]^−^ for α-viniferin and [C_60_H_41_N_5_O_15_]^−^ for α-viniferin-DPPH•) from the Total Ion Chromatogram, integrating the corresponding peaks. The scan range was fixed at 100–1600 Da.

### 3.8. Statistical Analysis

Each experiment was performed in triplicate and the data were recorded as means ± SD (standard deviation). The dose-response curves were plotted using Origin 6.0 professional software (OriginLab, Northampton, MA, USA). The IC_50_ value was defined as the final concentration of 50% radical inhibition (or relative reducing power). It was calculated by linear regression analysis and expressed as the mean ± SD (*n* = 3) [38]. The linear regression was analyzed by Origin 6.0 professional software. Statistical comparisons were made by one-way ANOVA to detect significant difference using SPSS 13.0 (SPSS Inc., Chicago, IL, USA) for windows. *p* < 0.05 was considered to be statistically significant.

## 4. Conclusions

To conclude, in both α-viniferin and caraphenol A, antioxidant reaction can proceed by redox-mediated mechanisms (especially ET and H^+^-transfer) as well as non-redox-mediated mechanisms (including Fe^2+^-chelating and RAF). The π-π conjugation of the exocyclic double bond in caraphenol A can greatly enhance the redox-mediated antioxidant mechanisms, and slightly strengthen the Fe^2+^-chelation mechanism. These factors result in the total antioxidant capacity of caraphenol A being much higher than that of α-viniferin.

## Supplementary Materials

The following are available online. Supplementary 1: Dose response curves; Supplementary 2: Original spectra of UPLC–ESI–Q–TOF–MS/MS analysis; Supplementary 3: Appearances and analysis certificates of α-viniferin and caraphenol A.

## Abbreviations

The following abbreviations are used in this manuscript:

ABTS	2,2′-azino-bis(3-ethylbenzo-thiazoline-6-sulfonic acid diammonium salt)
DPPH•	1,1-diphenyl-2-picryl-hydrazl
ET	electron transfer
FRAP	ferric-reducing antioxidant power
HAT	hydrogen atom transfer
HIV	human immunodeficiency virus
ROS	reactive oxygen species
RAF	radical adduct formation
SD	standard deviation
TPTZ	2,4,6-tris(2-pyridyl-s-triazine)
Trolox	(±)-6-hydroxyl-2,5,7,8-tetramethlychromane-2-carboxylic acid
