# Supplementary material for: π-π Conjugation Enhances Oligostilbene’s Antioxidant Capacity: Evidence from α-Viniferin and Caraphenol A
Source: Molecules. 2018 Mar 19;23(3):694. doi: 10.3390/molecules23030694 (PMC6017043; doi:10.3390/molecules23030694)
Supplement: Supplementary file 1 [file molecules-23-00694-s001.zip › Suppls/Suppl. 1 Dose response curves.docx]

**Supplemental Material-1 Dose response curves**

**π -π Conjugation Enhances Oligostilbene’s Antioxidant Capacity: Evidence from α-Viniferin and Caraphenol A**

**Xican Li ^1, 2, *,†^, Yulu Xie ^1, 2, †^**,**Hong Xie ^1, 2^**, **Jian Yang ^1^, and Dongfeng Chen ^3, 4, *^**

^1^ School of Chinese Herbal Medicine; xieyulu1900@163.com (Y.X.); xiehongxh1@163.com (H.X.); 1214640408@qq.com(J.Y.)

^2^ Innovative Research & Development Laboratory of TCM;

^3^ School of Basic Medical Science, Guangzhou University of Chinese Medicine;

^4^ The Research Center of Basic Integrative Medicine, Guangzhou University of Chinese Medicine. Waihuan East Road No. 232, Guangzhou Higher Education Mega Center, Guangzhou 510006, China.

^*^ Corresponding author. E-mail: [lixican@126. com](mailto:lixican@126.com)(X.L.); [chen888@gzucm.edu.cn](mailto:chen888@gzucm.edu.cn);

Tel.: +86-203-935-8076

**^†^** These authors contributed equally to this work.

**Note:** This Supplemental Material provides the Dose response curves and IC_50_ values. The data with underline are cited in Fig. 3 in the main text.


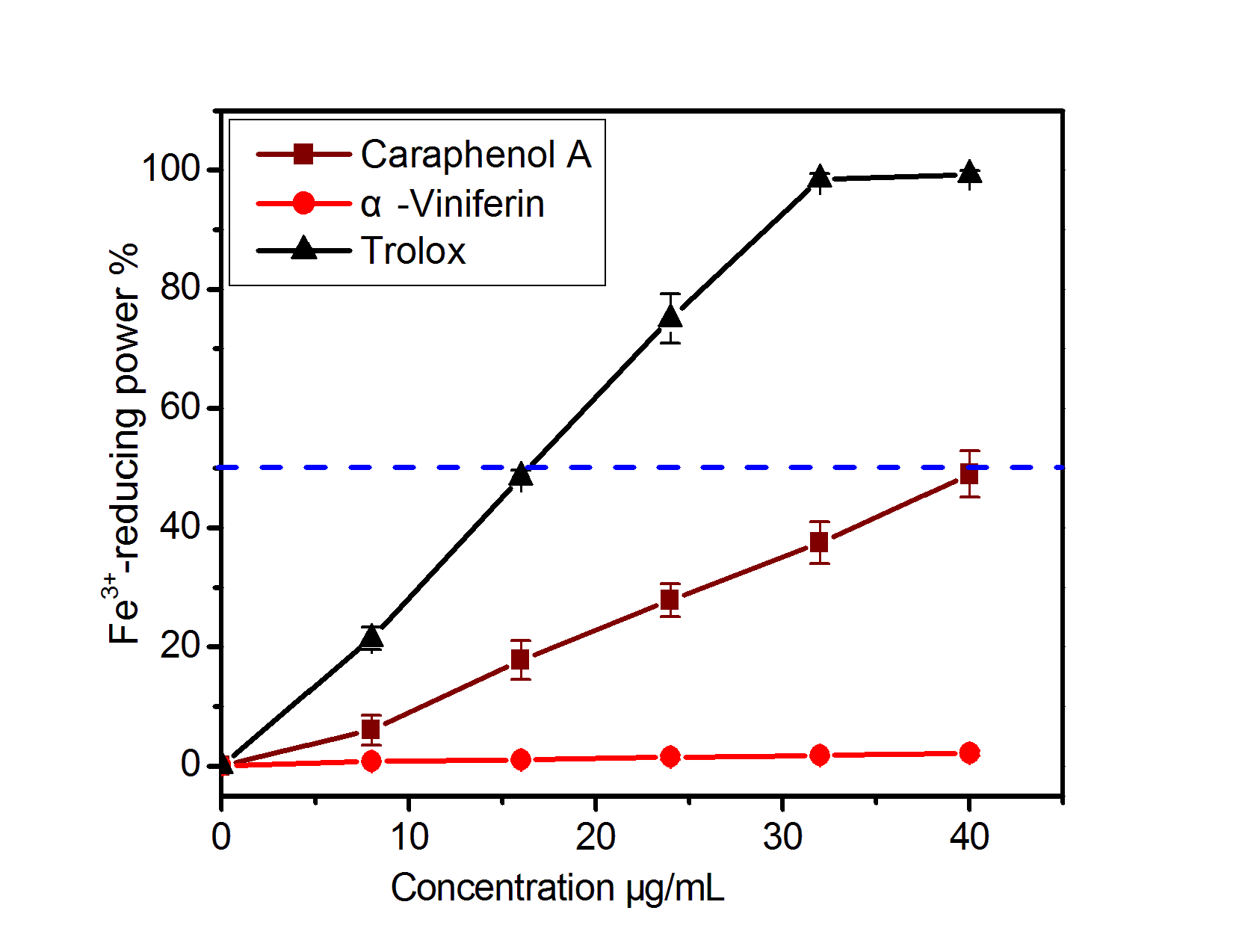


Figure S1**:** The dose response curves of α-viniferin and caraphenol A in FRAP assay. Trolox acts as the positive control. Each value is expressed as mean ± SD (n = 3).

Table. S1 The IC_50_ values of α-viniferin, caraphenol A, and Trolox in FRAP assay

|  | α-viniferin | caraphenol A | Trolox |
| --- | --- | --- | --- |
| IC_50_ value (μg/mL) | 1224.88±328.05 | 40.01±2.30 | 16.76±0.72 |
| IC_50_ value (μM) | 1804.75±483.35 ^c^ | 60.61±3.45^a^ | 66.97±2.89 ^b^ |

The IC_50_ value (μg/mL) was obtained from Figure S1. Each value is expressed as mean ± SD (n = 3). The IC_50_ values (μM) in the same row with different letters (a, b, or c) are significantly (p < 0.05) different among α-viniferin, caraphenol A, and Trolox.


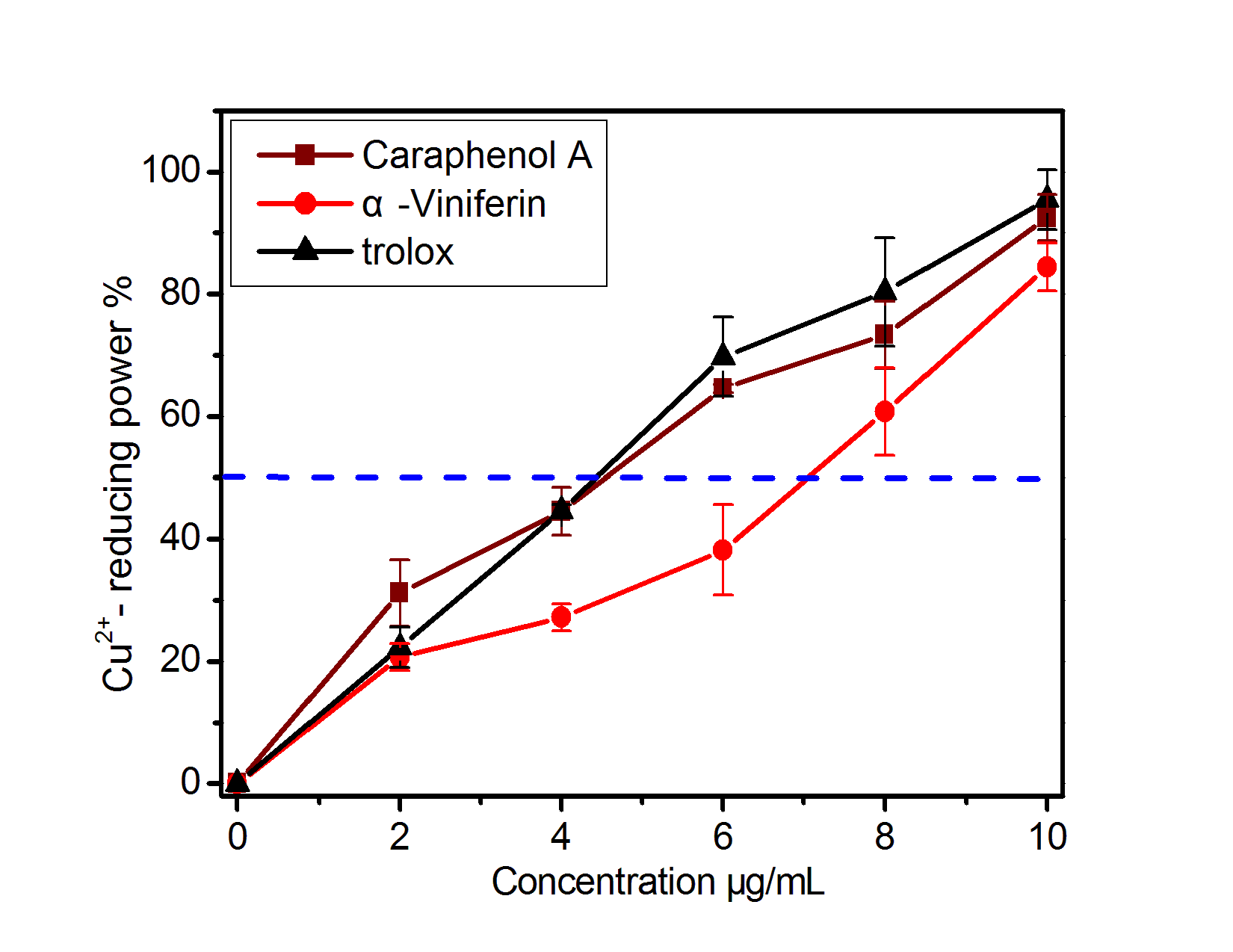


Figure S2**:** The dose response curves of α-viniferin and caraphenol A in Cu^2+^-reducing power (CUPRAC) assay. Each value is expressed as mean ± SD (n = 3).

Table. S2 The IC_50_ values of α-viniferin, caraphenol A, and Trolox in CUPRAC assay

|  | α-viniferin | caraphenol A | Trolox |
| --- | --- | --- | --- |
| IC_50_ value (μg/mL) | 6.47±0.40 | 3.74±0.27 | 4.64±0.15 |
| IC_50_ value (μM) | 9.53±0.59^b^ | 5.53±0.40^a^ | 18.54±0.59^c^ |

The IC_50_ value (μg/mL) was obtained from Figure S2. Each value is expressed as mean ± SD (n = 3). The IC_50_ values (μM) in the same row with different letters (a, b, or c) are significantly (p < 0.05) different among α-viniferin, caraphenol A, and Trolox.


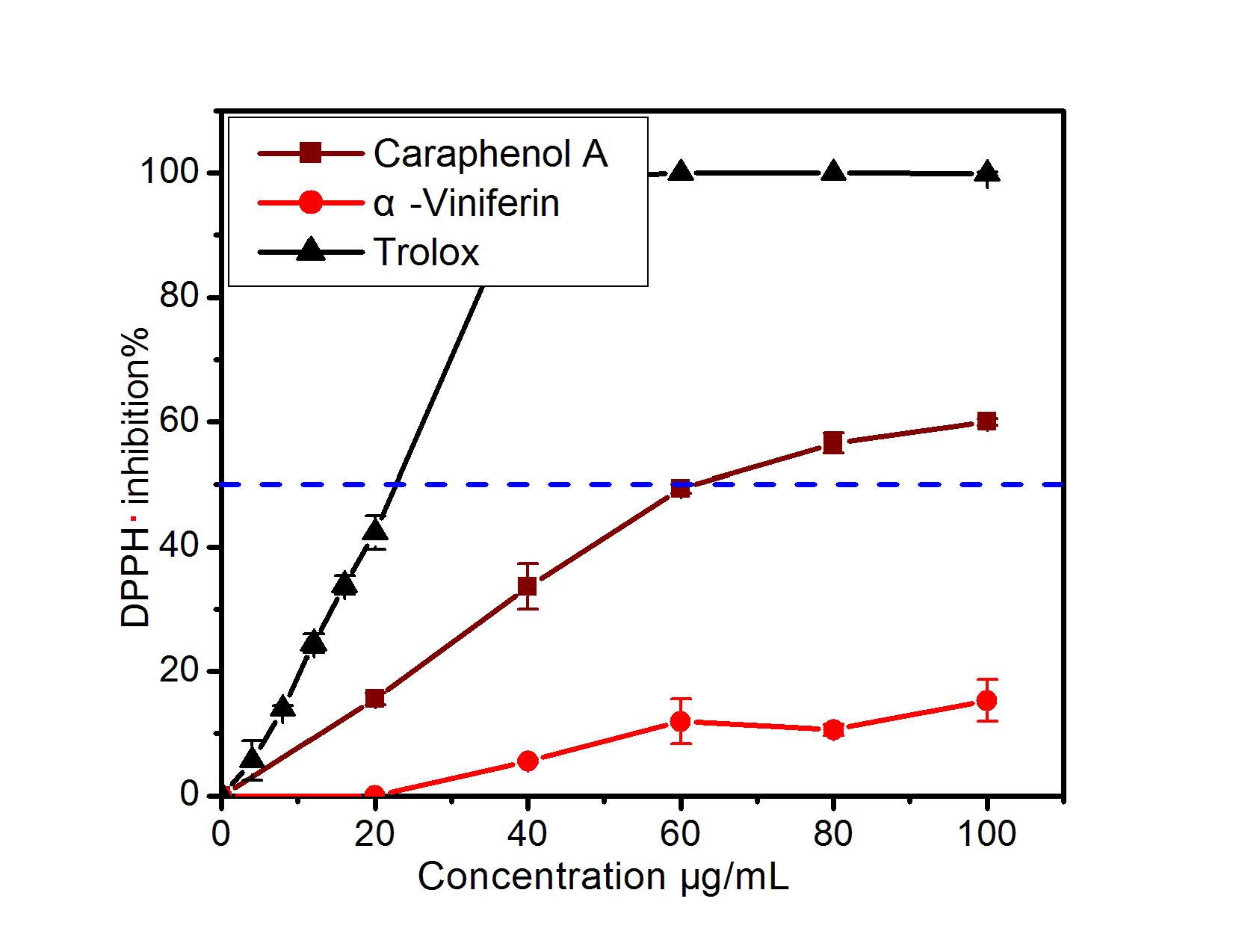


Figure S3**:** The dose response curves of α-viniferin and caraphenol A in DPPH•-scavenging assay. Each value is expressed as mean ± SD (n = 3).

Table. S3 The IC_50_ values of α-viniferin, caraphenol A, and Trolox in DPPH•-scavenging assay

|  | α-viniferin | caraphenol A | Trolox |
| --- | --- | --- | --- |
| IC_50_ value (μg/mL) | 340.67±72.31 | 72.49±0.49 | 22.84±0.74 |
| IC_50_ value (μM) | 501.94±106.55^b^ | 107.00±0.73^a^ | 91.28±231.49 ^a^ |

The IC_50_ value (μg/mL) was obtained from Figure S3. Each value is expressed as mean ± SD (n = 3). The IC_50_ values (μM) in the same row with different letters (a, b, or c) are significantly (p < 0.05) different among α-viniferin, caraphenol A, and Trolox.


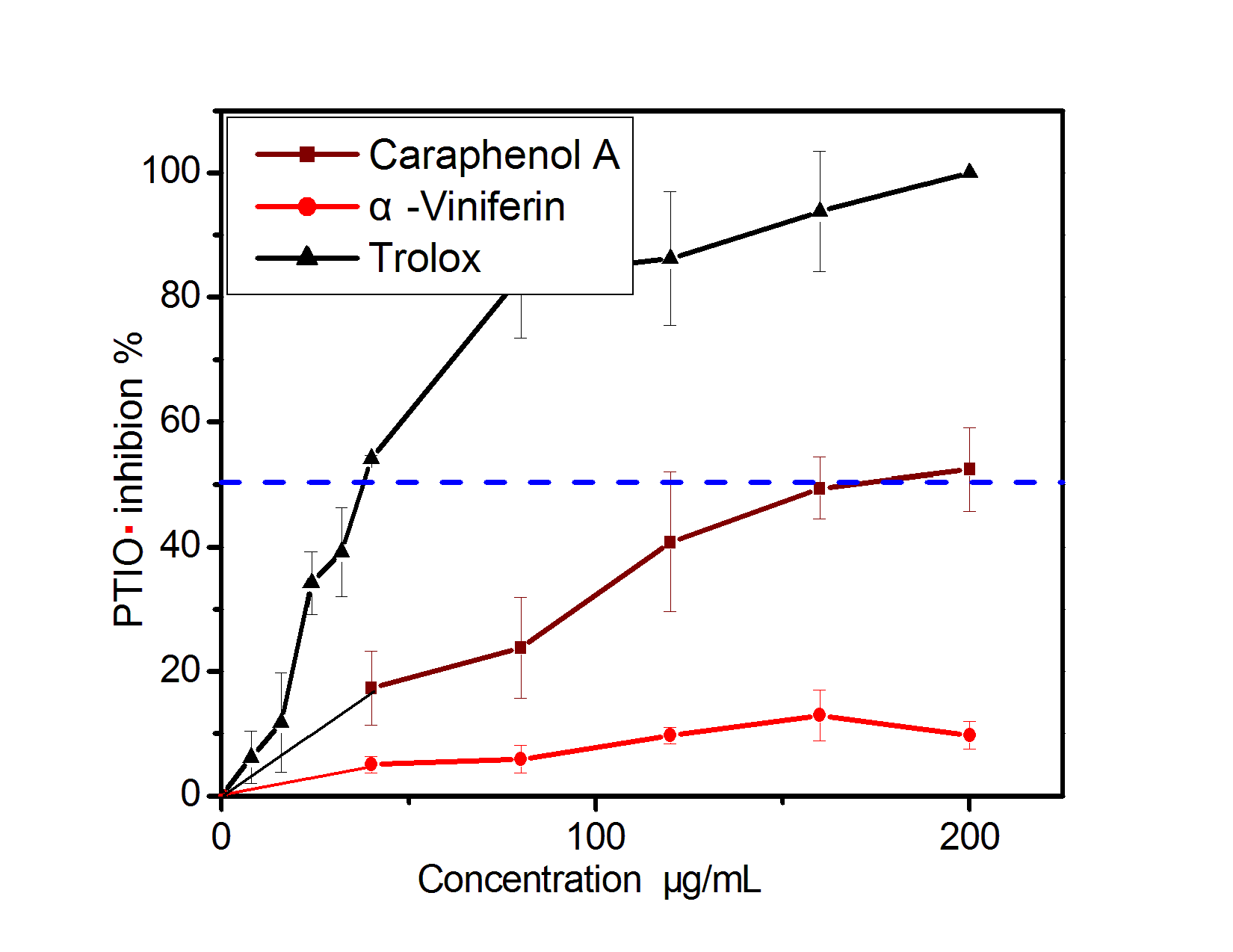


Figure S4**:** The dose response curves of α-viniferin and caraphenol A in PTIO•-scavenging assay. Each value is expressed as mean ± SD (n = 3).

Table. S4 The IC_50_ values of α-viniferin, caraphenol A, and Trolox PTIO•-scavenging assay

|  | α-viniferin | caraphenol A | Trolox |
| --- | --- | --- | --- |
| IC_50_ value (μg/mL) | 6.47±0.40 | 3.74±0.27 | 4.64±0.15 |
| IC_50_ value (μM) | 9.53±0.59^b^ | 5.53±0.40^a^ | 18.54±0.59^c^ |

The IC_50_ value (μg/mL) was obtained from Figure S4. Each value is expressed as mean ± SD (n = 3). The IC_50_ values (μM) in the same row with different letters (a, b, or c) are significantly (p < 0.05) different among α-viniferin, caraphenol A, and Trolox.
