# Supplementary material for: π-π Conjugation Enhances Oligostilbene’s Antioxidant Capacity: Evidence from α-Viniferin and Caraphenol A
Source: Molecules. 2018 Mar 19;23(3):694. doi: 10.3390/molecules23030694 (PMC6017043; doi:10.3390/molecules23030694)
Supplement: Supplementary file 1 [file molecules-23-00694-s001.zip › Suppls/Suppl. 2 HPLC-MS spectra Li.docx]

**Supplemental Material-2 Original HPLC-MS spectra**

**π -π Conjugation Enhances Oligostilbene’s Antioxidant Capacity: Evidence from α-Viniferin and Caraphenol A**

Xican Li ^1, 2, *,†^, Yulu Xie ^1, 2, †^, Hong Xie ^1, 2^, Jian Yang ^1^, and Dongfeng Chen ^3, 4, *^

^1^ School of Chinese Herbal Medicine; xieyulu1900@163.com (Y.X.); xiehongxh1@163.com (H.X.); [1214640408@qq.com](mailto:1214640408@qq.com) (J.Y.)

^2^ Innovative Research & Development Laboratory of TCM;

^3^ School of Basic Medical Science, Guangzhou University of Chinese Medicine;

^4^ The Research Center of Basic Integrative Medicine, Guangzhou University of Chinese Medicine. Waihuan East Road No. 232, Guangzhou Higher Education Mega Center, Guangzhou 510006, China.

^*^ Corresponding author. E-mail: [lixican@126. com](mailto:lixican@126.com) (X.L.); [lixc@gzucm.edu.cn](mailto:lixc@gzucm.edu.cn) (X.L.); [chen888@gzucm.edu.cn](mailto:chen888@gzucm.edu.cn) (D.C). Tel.: +86-203-935-8076

**^†^** These authors contributed equally to this work.

**Note:**

The supplemental materials provide the original spectra for Fig. 5 of the main text. In general, the reaction of α-viniferin with DPPH radical gave four RAF peaks at 3.653, 4.496, 4.811, and 6.479 min. However, caraphenol A with DPPH radical gave no RAF peaks.

In UPLC−ESI−Q−TOF−MS/MS analysis, the molecular ion peak is usually called primary MS spectra (molecular ion peaks). When the molecular ion peak is broken by ESI, it further gives rise to several fragments which are usually called secondary MS spectra, or MS/MS spectra.


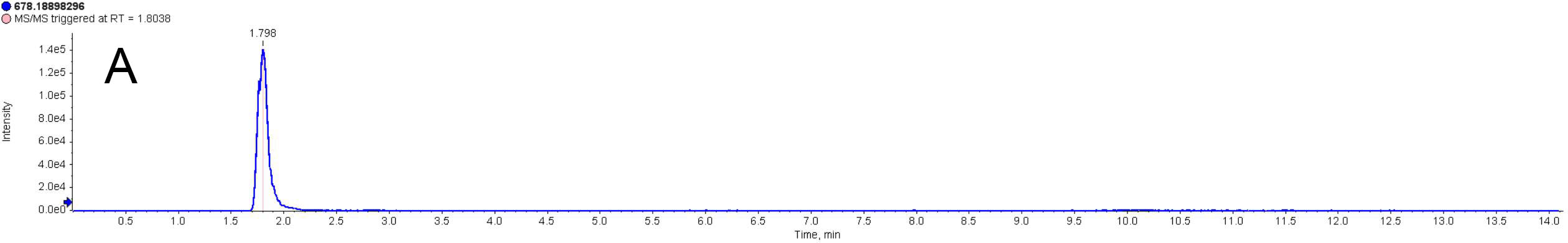

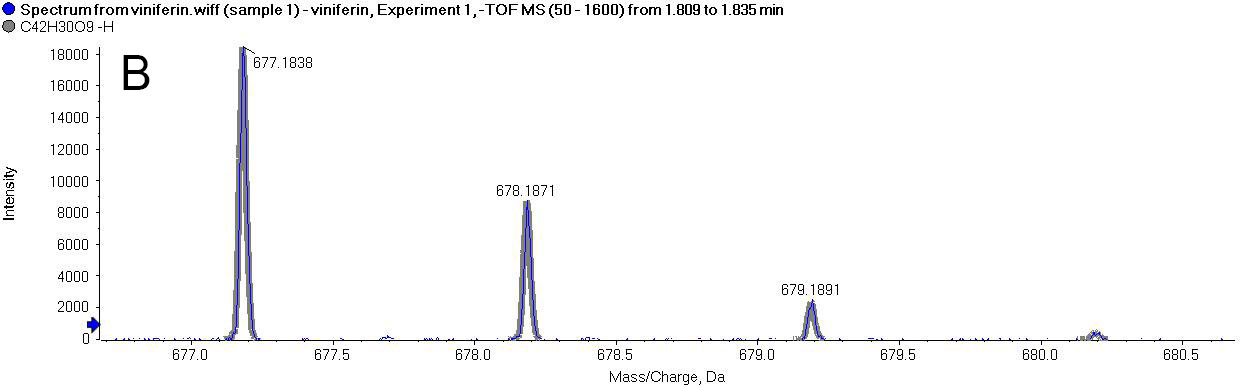

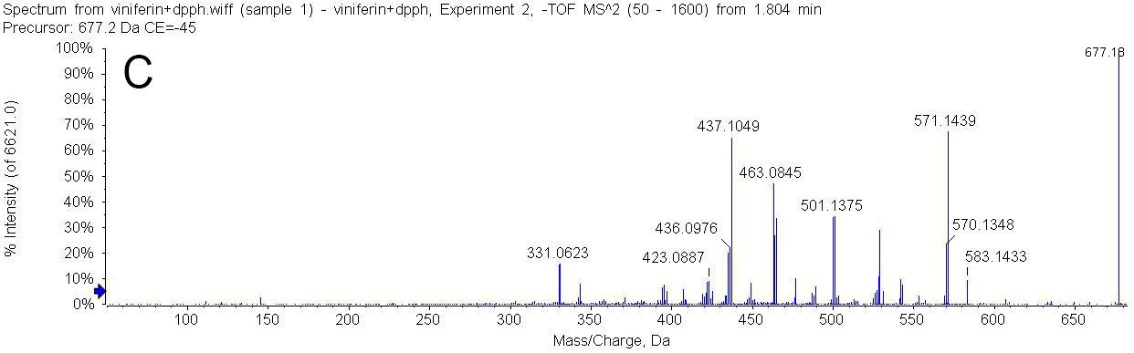


Fig. S 2.1 (A) Total ion chromatogram of α-viniferin standard extracted by corresponding chemical formula [C_42_H_30_O_9_-H]^-^; (B) Primary MS spectra (molecular ion peaks) of α-viniferin; (C) Secondary MS spectra of α-viniferin.

The determining conditions are detailed in Section 3.7 of main text.


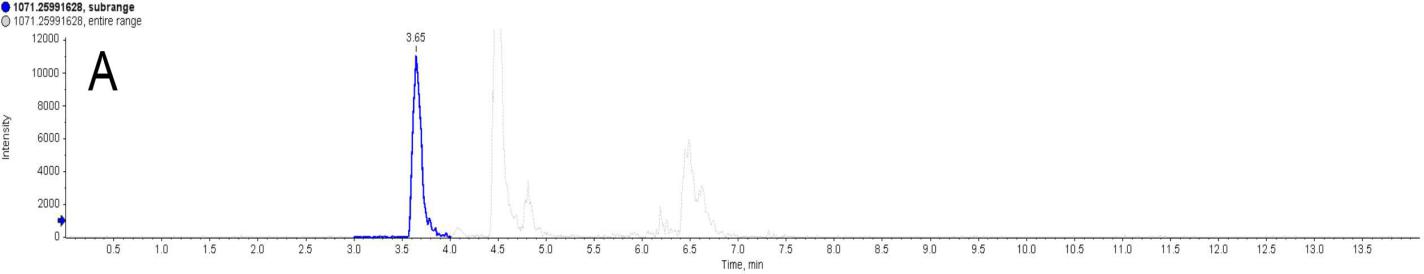

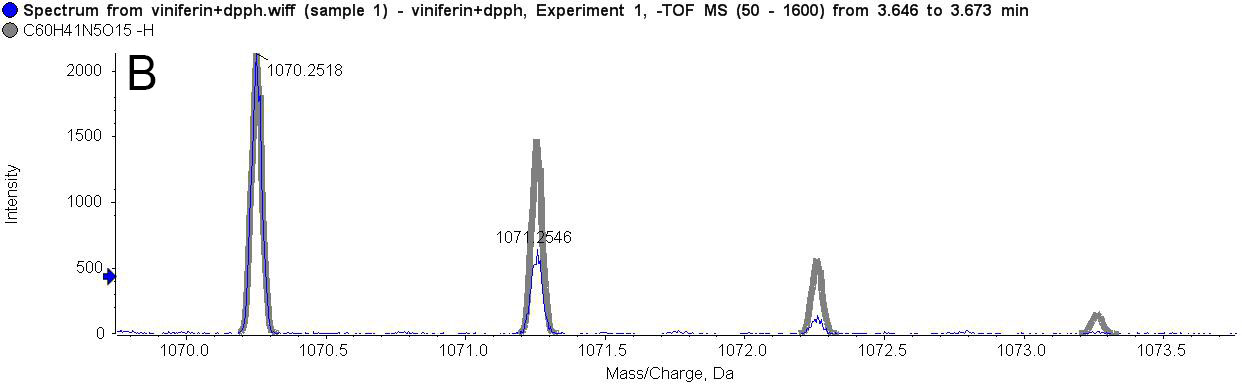


Fig. S 2.2 (A) [Ion chromatogram of](http://www.baidu.com/link?url=CF2kwqGsxR0oJevz0eMOkaKlSp5m3CP5azTuAPtPYQywgNd79FAccvr91aMdqMonPznhus0HQnaImc9imgKjcZGfDHgKz2a0BLH0fQFGHU8hiKdjXP7HKk5Oo13Brtbt)  reaction product of α-viniferin with DPPH• extracted by corresponding chemical formula [C_60_H_42_N_5_O_15_]. (B) Primary MS spectra (molecular ion peaks) of α-viniferin-DPPH

The peak was observed at 3.653 min. α-viniferin is C_42_H_30_O_9_; DPPH• is C_18_H_12_N_5_O_6._ Thus, [covalent](http://www.baidu.com/link?url=_NXpqH1Jy8-wYHBhYKz4OjCAE3JoN555TWPHLpcBZLLZRIaurfOQAmGBjgiSpGmKGQT_S1R2aAwmy80CmAIxxeLdfEfJ5extKEgaPUc1uAm) adduct should be C_60_H_42_N_5_O_15_, and the molecular weight should be 1070. The reaction was conducted by mixing acteoside and DPPH• (1:2, molar ratio). The reaction mixture was incubated for 24 h. However, the peak at 3.653 min is too weak to give rise to fragment peaks (Secondary MS spectra)

The determining conditions are detailed in Section 3.7 of main text.


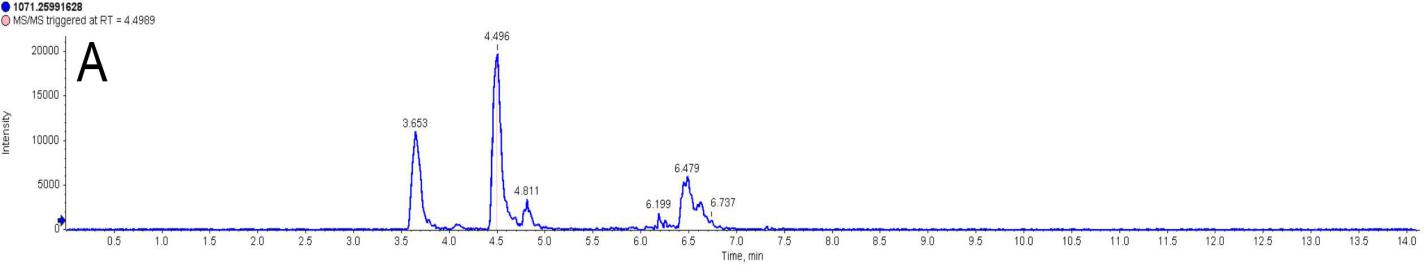

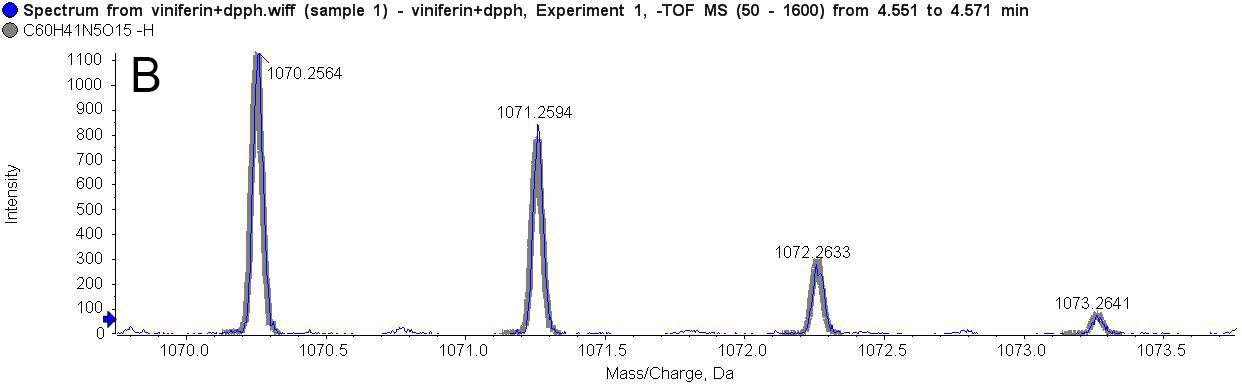

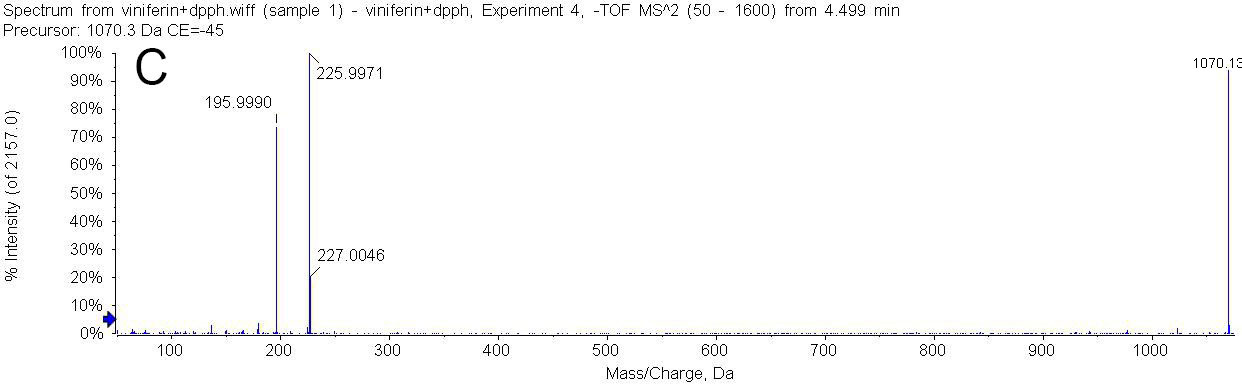


Fig. S 2.3 (A) [Ion chromatogram of](http://www.baidu.com/link?url=CF2kwqGsxR0oJevz0eMOkaKlSp5m3CP5azTuAPtPYQywgNd79FAccvr91aMdqMonPznhus0HQnaImc9imgKjcZGfDHgKz2a0BLH0fQFGHU8hiKdjXP7HKk5Oo13Brtbt)  reaction product of α-viniferin with DPPH• extracted by corresponding chemical formula [C_60_H_42_N_5_O_15_]. (B) Primary MS spectra (molecular ion peaks) of α-viniferin-DPPH. (C) Secondary MS spectra (molecular ion peaks) of α-viniferin-DPPH. The peak m/z 225 is thought to be a loss from DPPH moiety.

The peak was observed at 4.496 min. α-viniferin is C_42_H_30_O_9_; DPPH• is C_18_H_12_N_5_O_6._ Thus, [covalent](http://www.baidu.com/link?url=_NXpqH1Jy8-wYHBhYKz4OjCAE3JoN555TWPHLpcBZLLZRIaurfOQAmGBjgiSpGmKGQT_S1R2aAwmy80CmAIxxeLdfEfJ5extKEgaPUc1uAm) adduct should be C_60_H_42_N_5_O_15_, and the molecular weight should be 1070. The reaction was conducted by mixing acteoside and DPPH• (1:2, molar ratio). The reaction mixture was incubated for 24 h. However, the peak at 4.496 min further gave rise to fragment peaks (Secondary MS spectra, Fig. C).The determining conditions are detailed in Section 3.7 of main text.


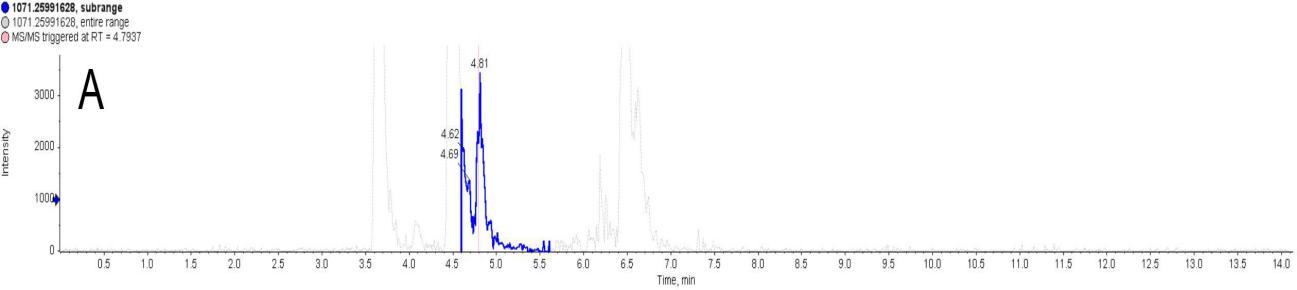

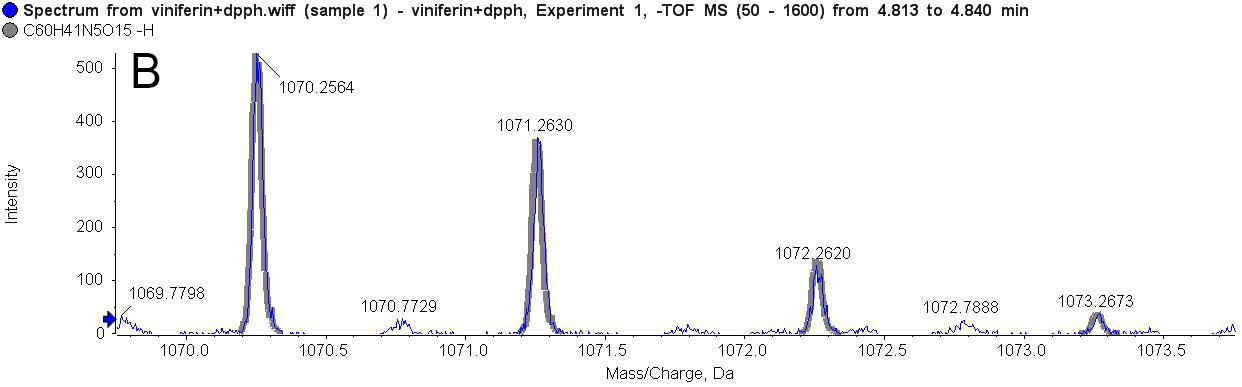

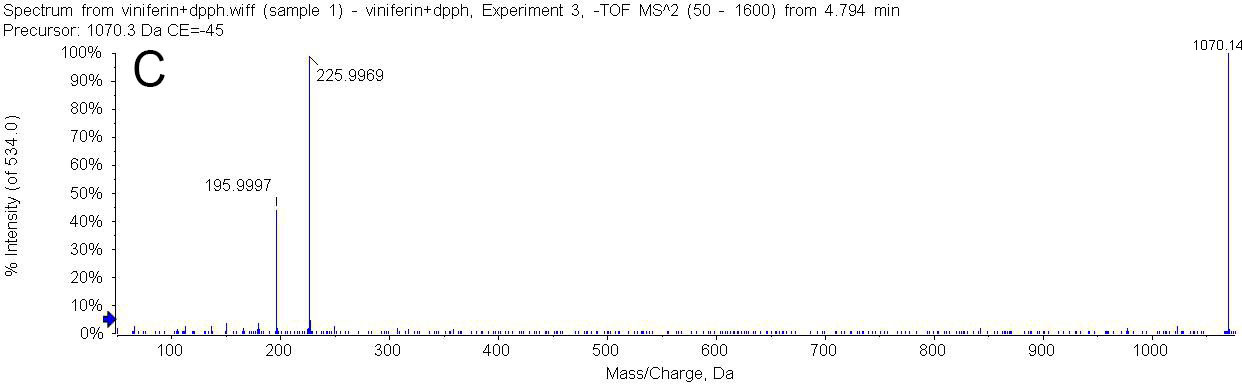


Fig. S 2.4 (A) [Ion chromatogram of](http://www.baidu.com/link?url=CF2kwqGsxR0oJevz0eMOkaKlSp5m3CP5azTuAPtPYQywgNd79FAccvr91aMdqMonPznhus0HQnaImc9imgKjcZGfDHgKz2a0BLH0fQFGHU8hiKdjXP7HKk5Oo13Brtbt)  reaction product of α-viniferin with DPPH• extracted by corresponding chemical formula [C_60_H_42_N_5_O_15_]. (B) Primary MS spectra (molecular ion peaks) of α-viniferin-DPPH. (C) Secondary MS spectra (molecular ion peaks) of α-viniferin-DPPH. The peak m/z 225 is thought to be a loss from DPPH moiety.

The peak was observed at 4.811 min. α-viniferin is C_42_H_30_O_9_; DPPH• is C_18_H_12_N_5_O_6._ Thus, [covalent](http://www.baidu.com/link?url=_NXpqH1Jy8-wYHBhYKz4OjCAE3JoN555TWPHLpcBZLLZRIaurfOQAmGBjgiSpGmKGQT_S1R2aAwmy80CmAIxxeLdfEfJ5extKEgaPUc1uAm) adduct should be C_60_H_42_N_5_O_15_, and the molecular weight should be 1070. The reaction was conducted by mixing acteoside and DPPH• (1:2, molar ratio). The reaction mixture was incubated for 24 h. However, the peak at 4.811 min further gave rise to fragment peaks (Secondary MS spectra, Fig. C).The determining conditions are detailed in Section 3.7 of main text.


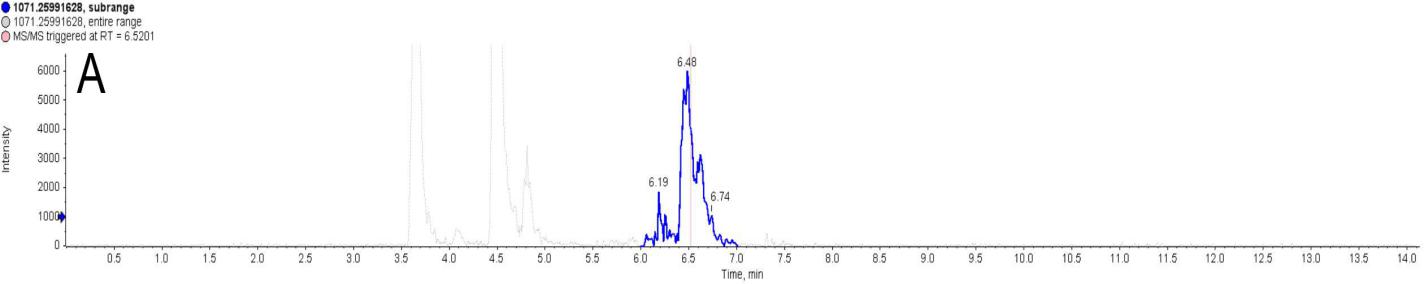

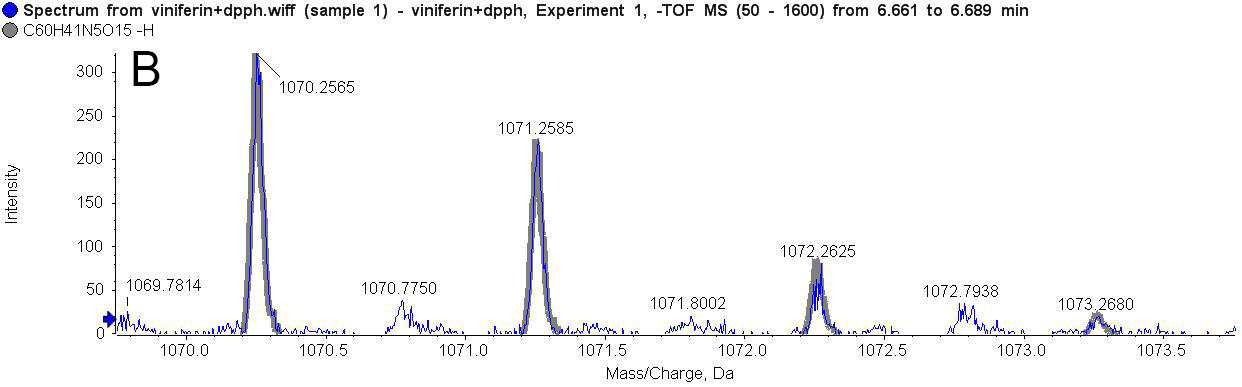

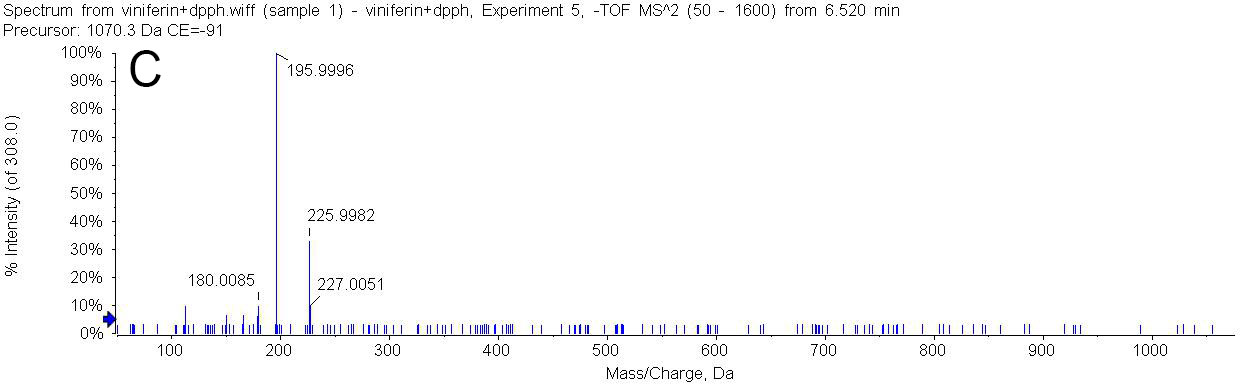


Fig. S 2.5 (A) [Ion chromatogram of](http://www.baidu.com/link?url=CF2kwqGsxR0oJevz0eMOkaKlSp5m3CP5azTuAPtPYQywgNd79FAccvr91aMdqMonPznhus0HQnaImc9imgKjcZGfDHgKz2a0BLH0fQFGHU8hiKdjXP7HKk5Oo13Brtbt)  reaction product of α-viniferin with DPPH• extracted by corresponding chemical formula [C_60_H_42_N_5_O_15_]. (B) Primary MS spectra (molecular ion peaks) of α-viniferin-DPPH. (C) Secondary MS spectra (molecular ion peaks) of α-viniferin-DPPH. The peak m/z 225 is thought to be a loss from DPPH moiety.

The peak was observed at 6.479 min. α-Viniferin is C_42_H_30_O_9_; DPPH• is C_18_H_12_N_5_O_6._ Thus, [covalent](http://www.baidu.com/link?url=_NXpqH1Jy8-wYHBhYKz4OjCAE3JoN555TWPHLpcBZLLZRIaurfOQAmGBjgiSpGmKGQT_S1R2aAwmy80CmAIxxeLdfEfJ5extKEgaPUc1uAm) adduct should be C_60_H_42_N_5_O_15_, and the molecular weight should be 1070. The reaction was conducted by mixing acteoside and DPPH• (1:2, molar ratio). The reaction mixture was incubated for 24 h. However, the peak at 6.479 min further gave rise to fragment peaks (Secondary MS spectra, Fig. C).The determining conditions are detailed in Section 3.7 of main text.

_
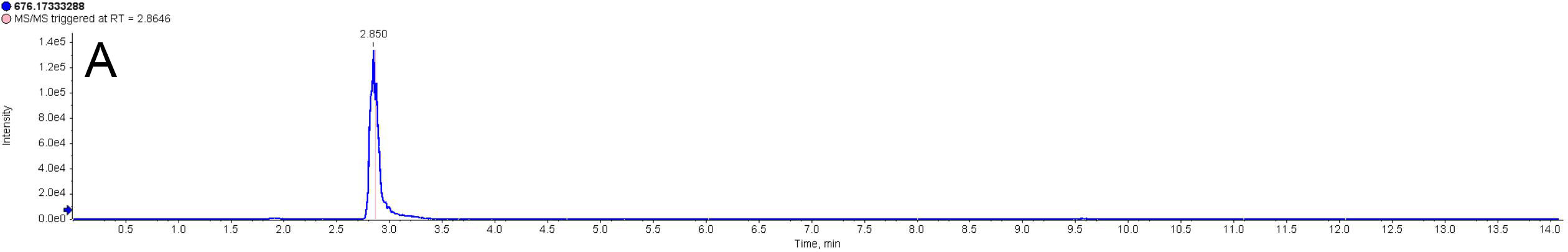
_


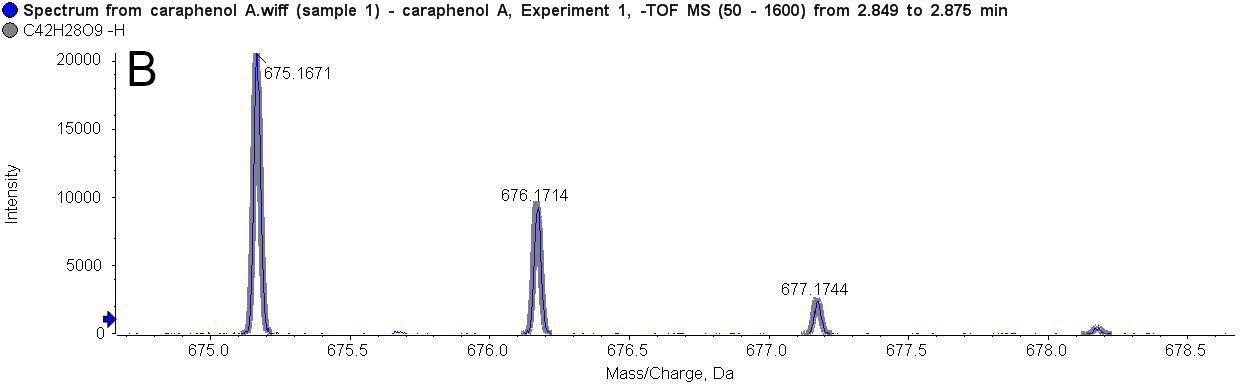

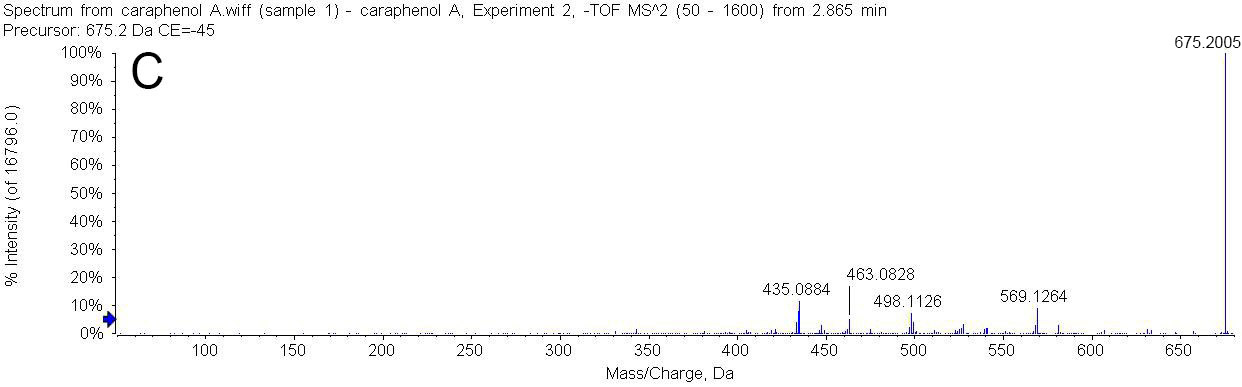


Fig. S 2.6 (A)Total ion chromatogram of caraphenol A standard when the formula [C_42_H_28_O_9_] was extracted; (B) Primary MS spectra (molecular ion peaks) of caraphenol A; (C) Secondary MS spectra of caraphenol A.

The peak was observed at 2.850 min. However, the peak at 2.850 min further gave rise to fragment peaks (Secondary MS spectra, Fig. C).The determining conditions are detailed in Section 3.7 of main text.

Fig. S 2.7 [Ion chromatogram of](http://www.baidu.com/link?url=CF2kwqGsxR0oJevz0eMOkaKlSp5m3CP5azTuAPtPYQywgNd79FAccvr91aMdqMonPznhus0HQnaImc9imgKjcZGfDHgKz2a0BLH0fQFGHU8hiKdjXP7HKk5Oo13Brtbt)  reaction product of caraphenol A with DPPH• extracted by [C_60_H_40_N_5_O_15_].

Caraphenol A is C_42_H_28_O_9_; DPPH• is C_18_H_12_N_5_O_6._ Thus, [covalent](http://www.baidu.com/link?url=_NXpqH1Jy8-wYHBhYKz4OjCAE3JoN555TWPHLpcBZLLZRIaurfOQAmGBjgiSpGmKGQT_S1R2aAwmy80CmAIxxeLdfEfJ5extKEgaPUc1uAm) adduct should be C_60_H_40_N_5_O_15_. However, no corresponding peak was found. All these peaks are noise.

The determining conditions are detailed in Section 3.7 of main text.
