# Supplementary material for: π-π Conjugation Enhances Oligostilbene’s Antioxidant Capacity: Evidence from α-Viniferin and Caraphenol A
Source: Molecules. 2018 Mar 19;23(3):694. doi: 10.3390/molecules23030694 (PMC6017043; doi:10.3390/molecules23030694)
Supplement: Supplementary file 1 [file molecules-23-00694-s001.zip › Suppls/Suppl. 3 Appearances & analysis certificates of α-viniferin and caraphenol A.pdf]

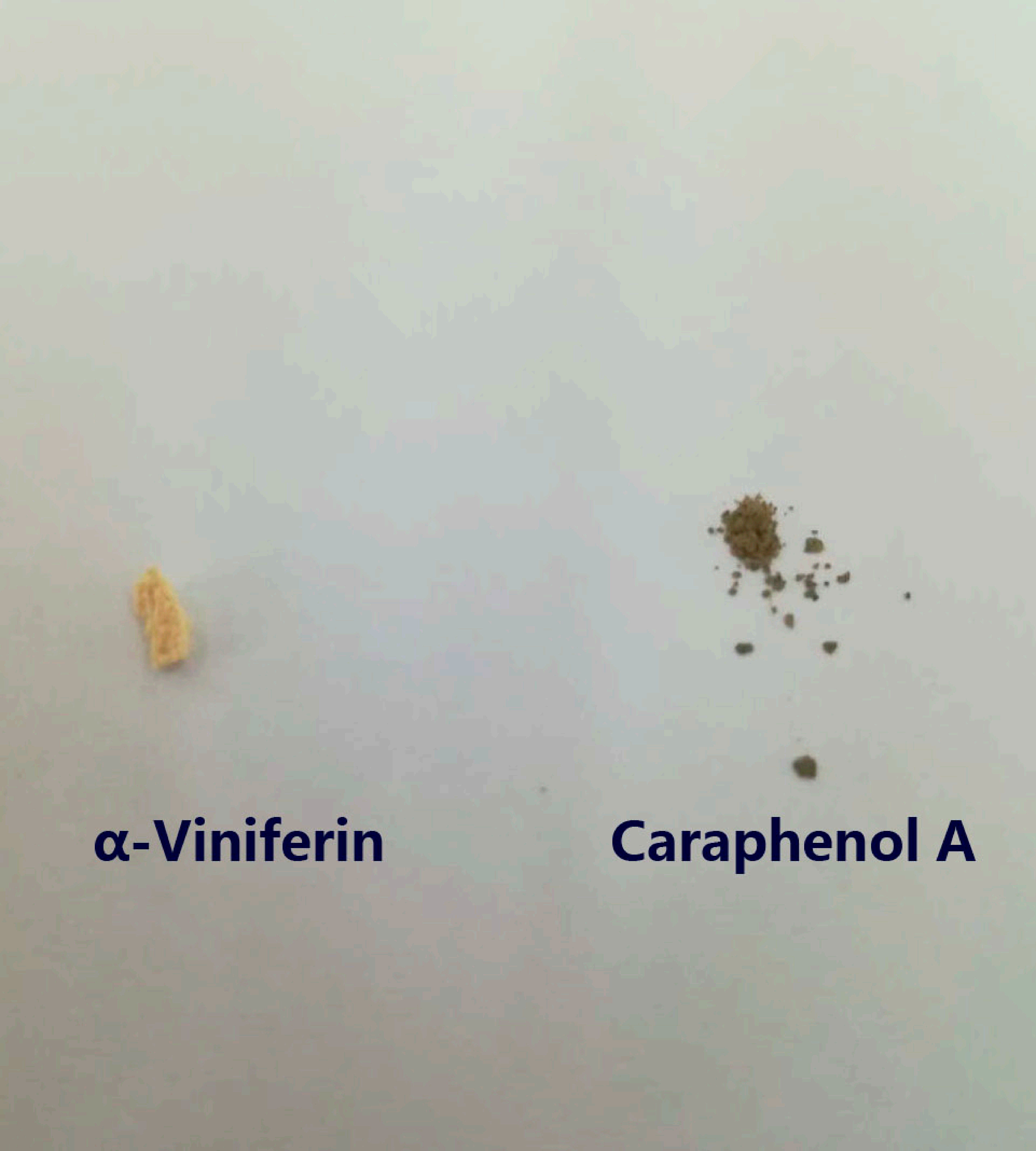
α-Viniferin
Caraphenol A

西力生物
BBP BioBioPha
Natural Product Library for Drug Discovery

CERTIFICATE OF ANALYSIS

BBP No.: BBP00225

CAS No.: 62218-13-7

Chemical Name: α-Viniferin

Molecular Formula: $C_{42}H_{30}O_9$

Structure:

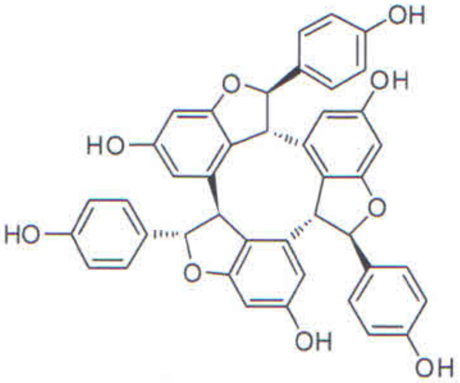

Purity: 97%

Appearance: Powder

Solvent: Acetone, methanol

Exact Weight: 5.1 mg

Storage: Store in a dark place under the temperature of 0−4 °C

Intended Use: For laboratory use only

Reference: R. J. Pryce, et al., Phytochemistry, 1977, 16, 1452-1454

Warm Notice: When publishing, please cite as: ***chemical name*** was purchased from BioBioPha Co., Ltd. (Kunming, China)

Characterization Data Summary

Analytical Test	Results
Identification by ^{1}H-NMR	Consistent with the above structure
Purity tested by HPLC, ^{1}H-NMR	97%

Authorized Signature: BioBioPha Co., Ltd. 2018.01.19 PASS

Date:

Tel.: +86 871 6521 7109 Fax: +86 871 6521 5563 E-mail: sales@mail.biobiopha.com

西力生物
BBP BioBioPha

Natural Product Library for Drug Discovery

PRODUCT QUALITY REPORT

Product Number: BBP00225

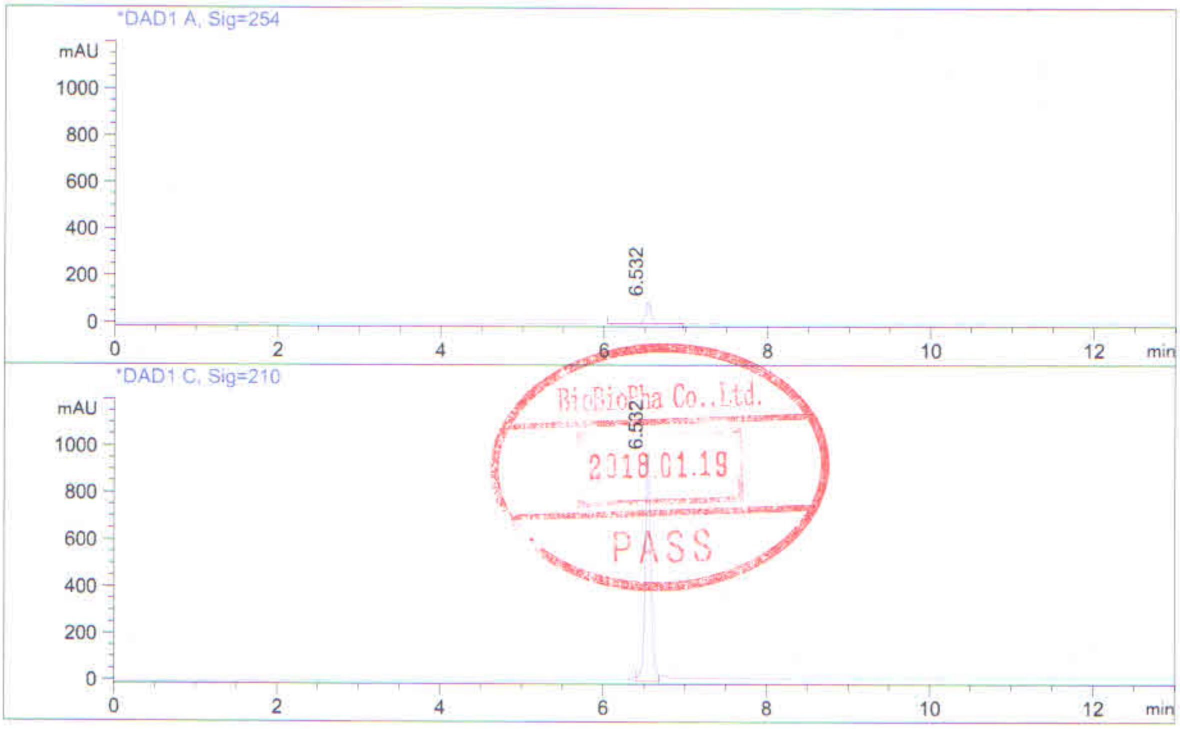

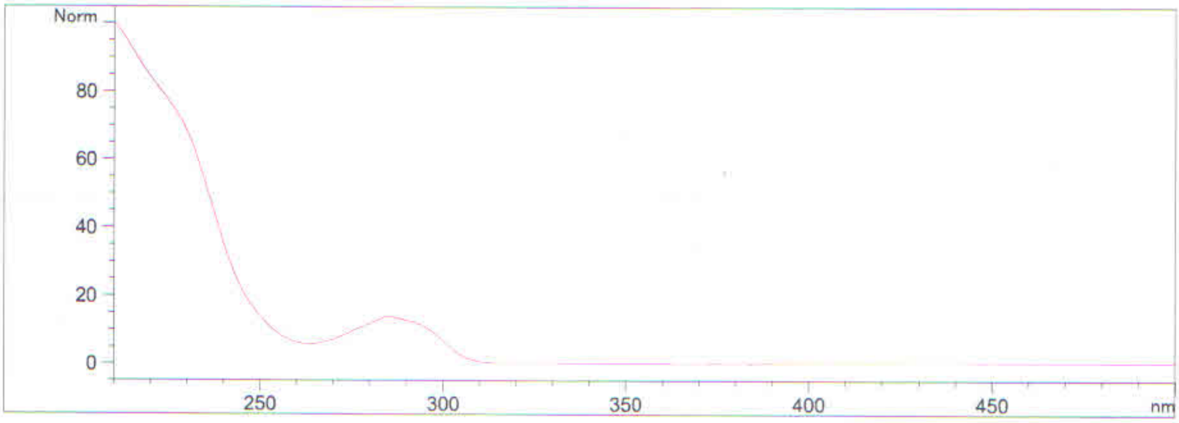

Agilent 1200 series HPLC system
Extend-C18 column (5 μm, 4.6 × 150 mm)
20%→100% MeOH in H_2O over 8.0 min followed by 100% MeOH to 13.0 min
1.0 ml/min, 25°C

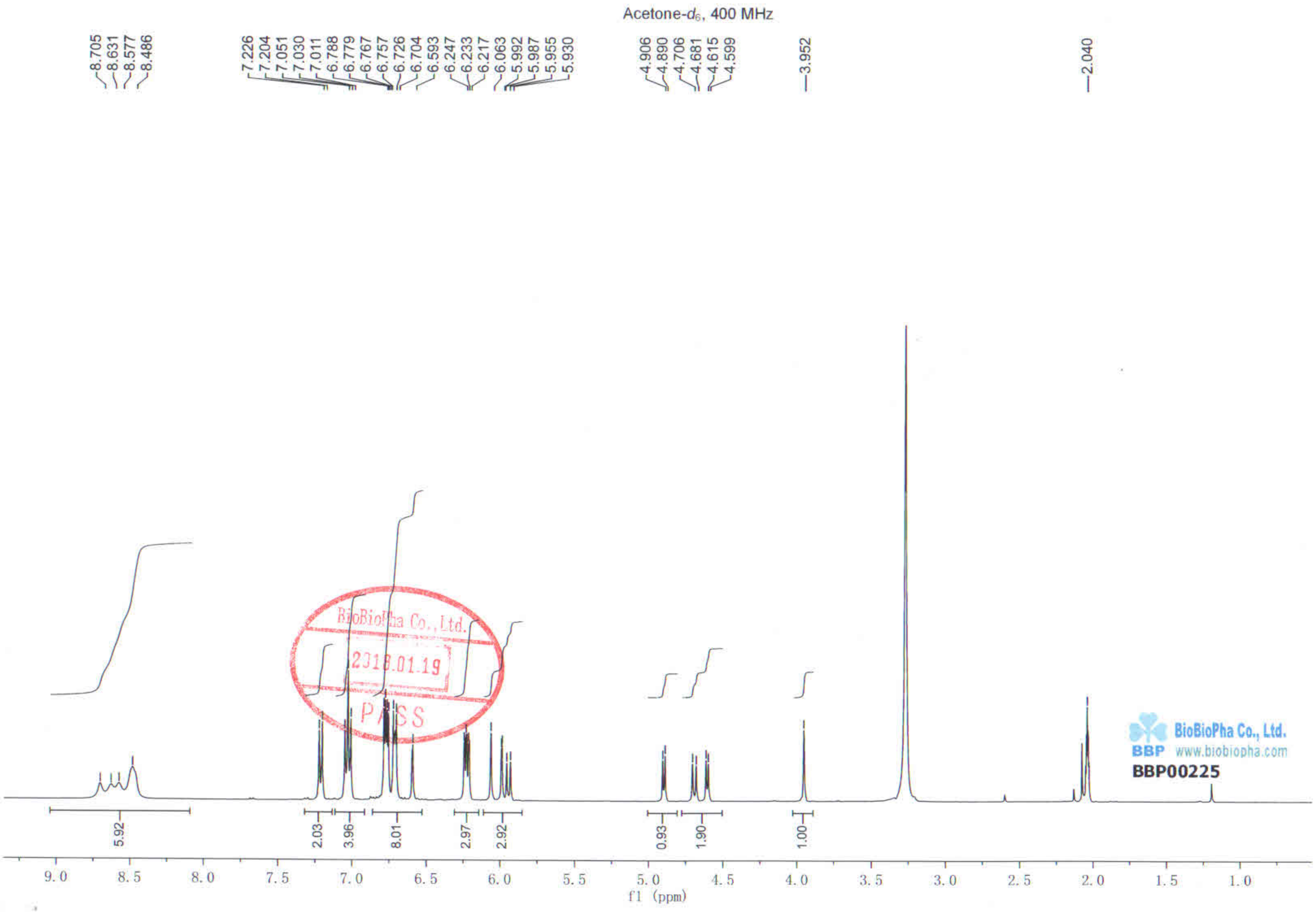
Acetone-d_6, 400 MHz
8.705 8.631 8.577 8.486
7.226 7.204 7.051 7.030 7.011 6.788 6.779 6.767 6.757 6.726 6.704 6.593 6.247 6.233 6.217 6.063 5.992 5.987 5.955 5.930
4.906 4.890 4.706 4.681 4.615 4.599
3.952
2.040
5.92 2.03 3.96 8.01 2.97 2.92 0.93 1.90 1.00
9.0 8.5 8.0 7.5 7.0 6.5 6.0 5.5 5.0 4.5 4.0 3.5 3.0 2.5 2.0 1.5 1.0
f1 (ppm)
BioBioPha Co., Ltd.
2018.01.19
PASS
BioBioPha Co., Ltd.
BBP www.biobiopha.com
BBP00225

西力生物
BBP BioBioPha

Natural Product Library for Drug Discovery

CERTIFICATE OF ANALYSIS

BBP No.: BBP00255

CAS No.: 354553-35-8

Chemical Name: Caraphenol A

Molecular Formula: $C_{42}H_{28}O_9$

Structure:

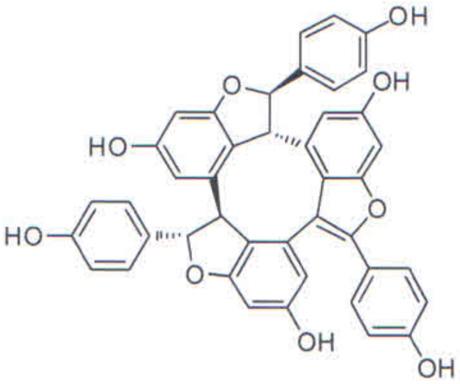

Purity: 97%

Appearance: Powder

Solvent: Acetone, methanol

Exact Weight: 5.1 mg

Storage: Store in a dark place under the temperature of 0−4 °C

Intended Use: For laboratory use only

Reference: H. F. Luo, et al., Tetrahedron, 2001, 57, 4849-4854

Warm Notice: When publishing, please cite as: ***chemical name*** was purchased from BioBioPha Co., Ltd. (Kunming, China)

Characterization Data Summary

Analytical Test	Results
Identification by ^{1}H-NMR	Consistent with the above structure
Purity tested by HPLC, ^{1}H-NMR	97%

Authorized Signature: BioBioPha Co., Ltd. 2018.01.19 PASS

Date:

Tel.: +86 871 6521 7109 Fax: +86 871 6521 5563 E-mail: sales@mail.biobiopha.com

西力生物
BBP BioBioPha

Natural Product Library for Drug Discovery

PRODUCT QUALITY REPORT

Product Number: BBP00255

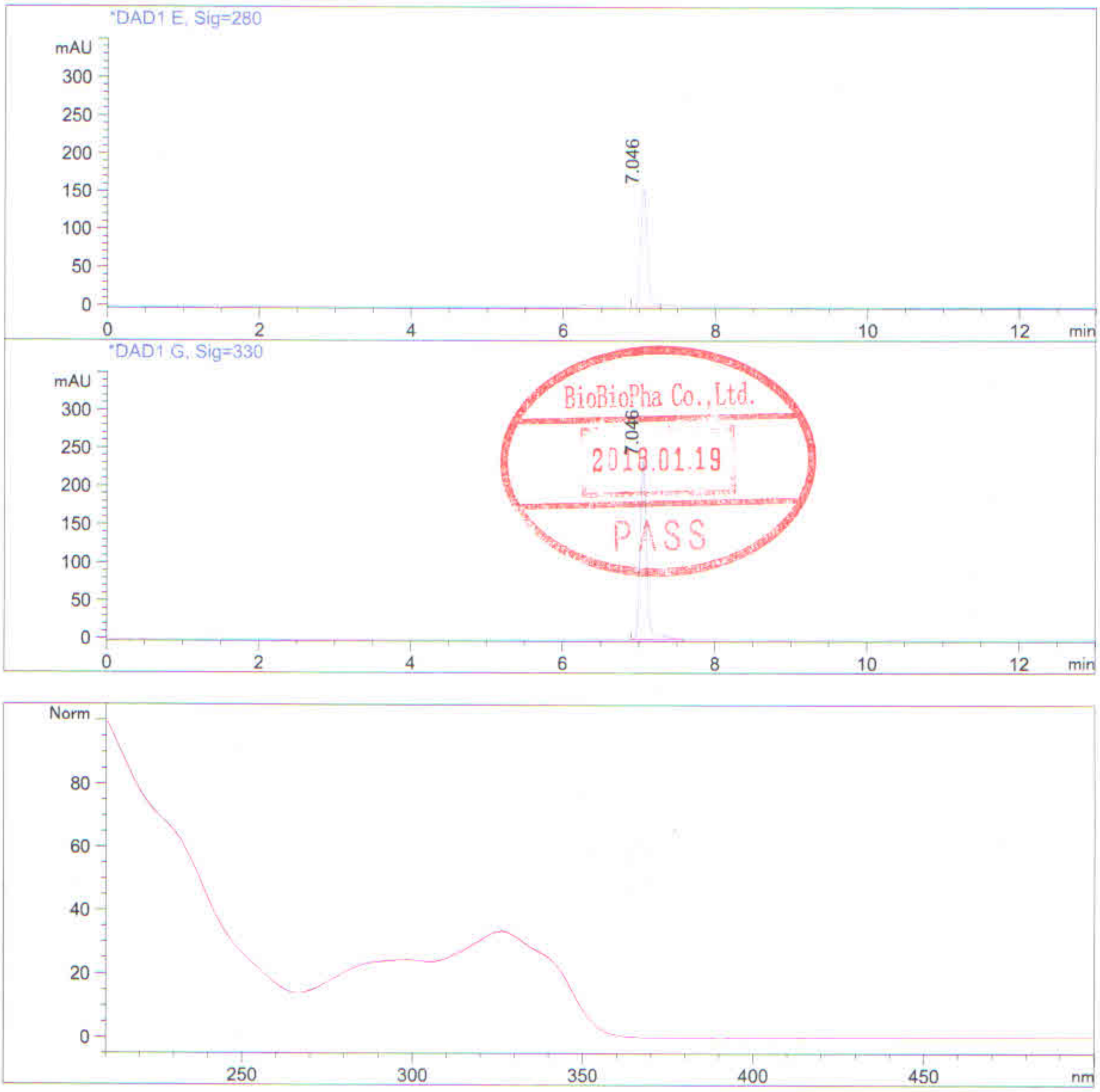

Agilent 1200 series HPLC system
Thermo Hypersil BDS C18 column (5 µm, 4.6 × 150 mm)
20%→100% MeOH in H_2O over 8.0 min followed by 100% MeOH to 13.0 min
1.0 ml/min, 25°C

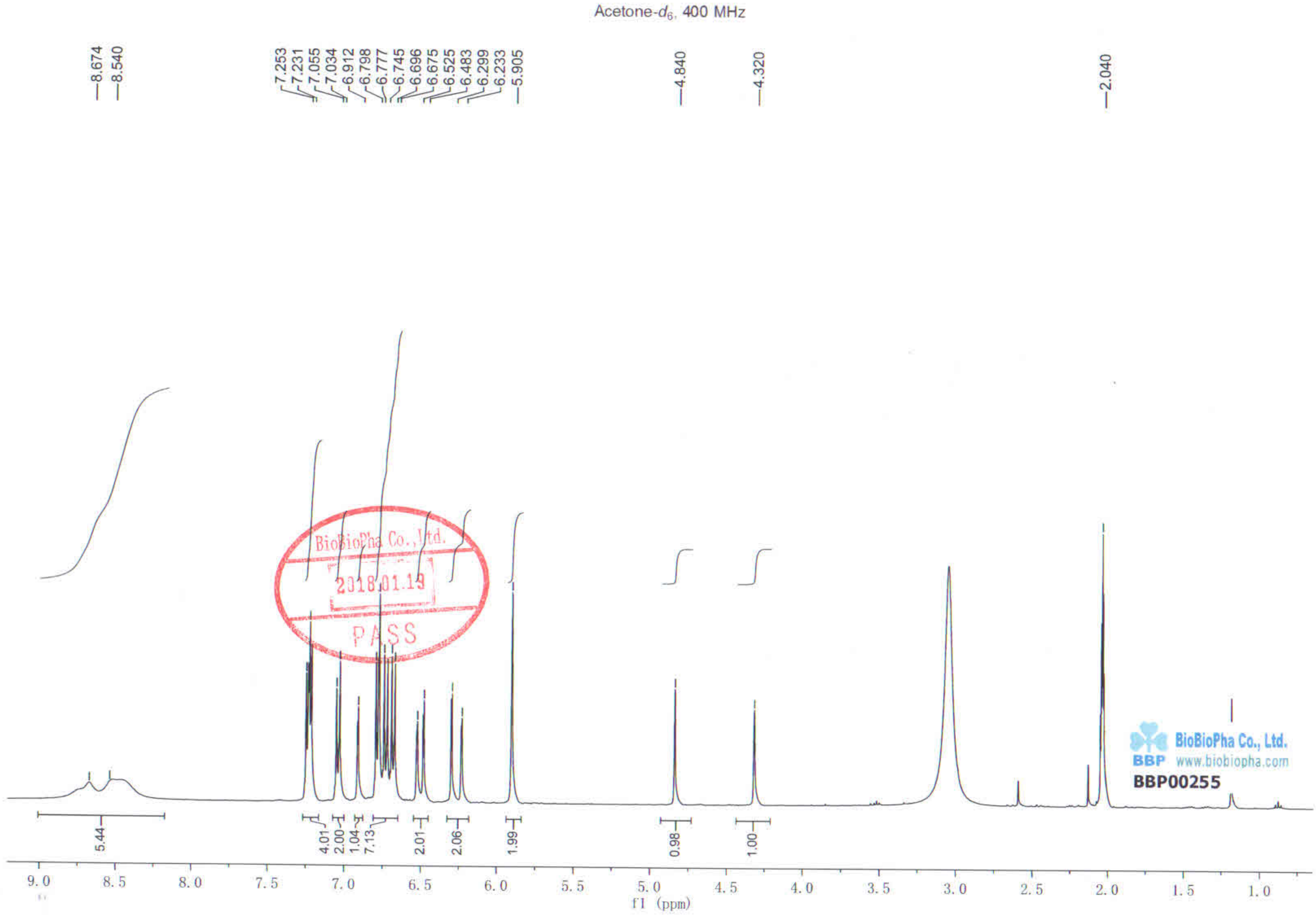

Acetone-d_6, 400 MHz
8.674
8.540
7.253
7.231
7.055
7.034
6.912
6.798
6.777
6.745
6.696
6.675
6.525
6.483
6.299
6.233
5.905
4.840
4.320
2.040
5.44
4.01
2.00
1.04
7.13
2.01
2.06
1.99
0.98
1.00
9.0
8.5
8.0
7.5
7.0
6.5
6.0
5.5
5.0
4.5
4.0
3.5
3.0
2.5
2.0
1.5
1.0
f1 (ppm)
BioBioPha Co., Ltd.
2018.01.19
PASS
BioBioPha Co., Ltd.
BBP www.biobiopha.com
BBP00255
