# Supplementary material for: π-π Conjugation Enhances Oligostilbene’s Antioxidant Capacity: Evidence from α-Viniferin and Caraphenol A
Source: Molecules. 2018 Mar 19;23(3):694. doi: 10.3390/molecules23030694 (PMC6017043; doi:10.3390/molecules23030694)
Supplement: Supplementary file 1 [file molecules-23-00694-s001.zip › Suppls/~WRL3156.tmp]

**Supplemental Material-1**

**π -π Conjugation Enhances Oligostilbene’s Antioxidant Capacity: Evidence from α-Viniferin and Caraphenol A**

**Xican Li ^1, 2, *,†^, Yulu Xie ^1, 2, †^**,**Hong Xie ^1, 2^**, **Jian Yang ^1^, and Dongfeng Chen ^3, 4, *^**

^1^ School of Chinese Herbal Medicine; xieyulu1900@163.com (Y.X.); xiehongxh1@163.com (H.X.); 1214640408@qq.com(J.Y.)

^2^ Innovative Research & Development Laboratory of TCM;

^3^ School of Basic Medical Science, Guangzhou University of Chinese Medicine;

^4^ The Research Center of Basic Integrative Medicine, Guangzhou University of Chinese Medicine. Waihuan East Road No. 232, Guangzhou Higher Education Mega Center, Guangzhou 510006, China.

^*^ Corresponding author. E-mail: [lixican@126. com](mailto:lixican@126.com)(X.L.); [chen888@gzucm.edu.cn](mailto:chen888@gzucm.edu.cn);

Tel.: +86-203-935-8076

**^†^** These authors contributed equally to this work.

**Note:** This Supplemental Material provides the original data of Fig. 3 in the main text. All data with underline are mentioned in **Tab. 1**.


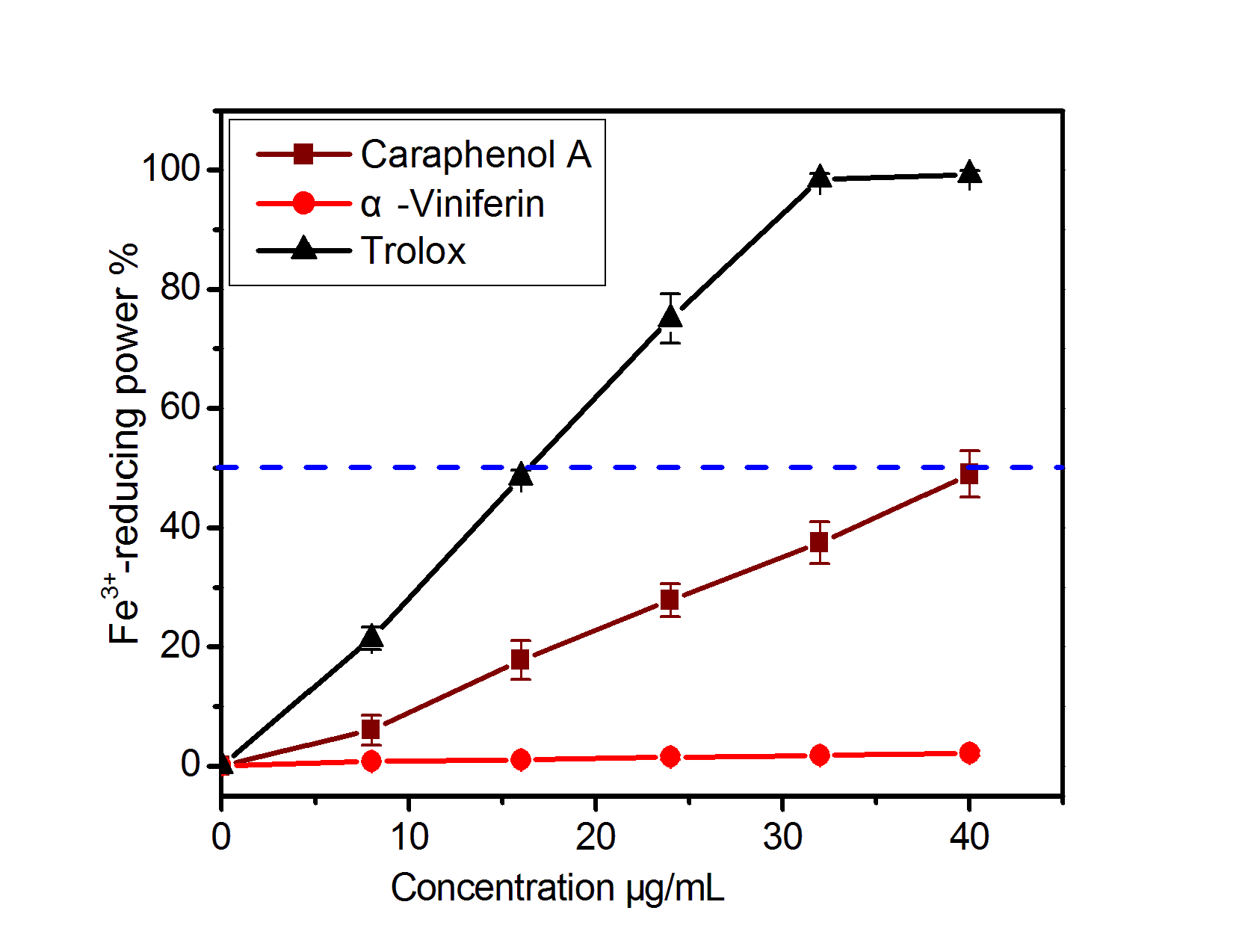


Figure S1**:** The dose response curves of α-viniferin and caraphenol A in FRAP assay. Trolox acts as the positive control. Each value is expressed as mean ± SD (n = 3).

Table. S1 The IC_50_ values of α-viniferin, caraphenol A, and Trolox in FRAP assay (μg/mL)

|  | α-viniferin | caraphenol A | Trolox |
| --- | --- | --- | --- |
| IC_50_ value (μg/mL) | 610.00 | 600.00 | 600.00 |
| IC_50_ value (μM) | 2410.00 | 2410.00 | 2430.00 |

IC_50_ value was defined as the concentration of 50% superoxide anion radical inhibition and calculated by linear regression which was analyzed by Origin 6.0 professional software. Means values with different superscripts in the same column are significantly different (*p<*0.05). Means values with different superscripts in the same column are significantly different (*p<*0.05).


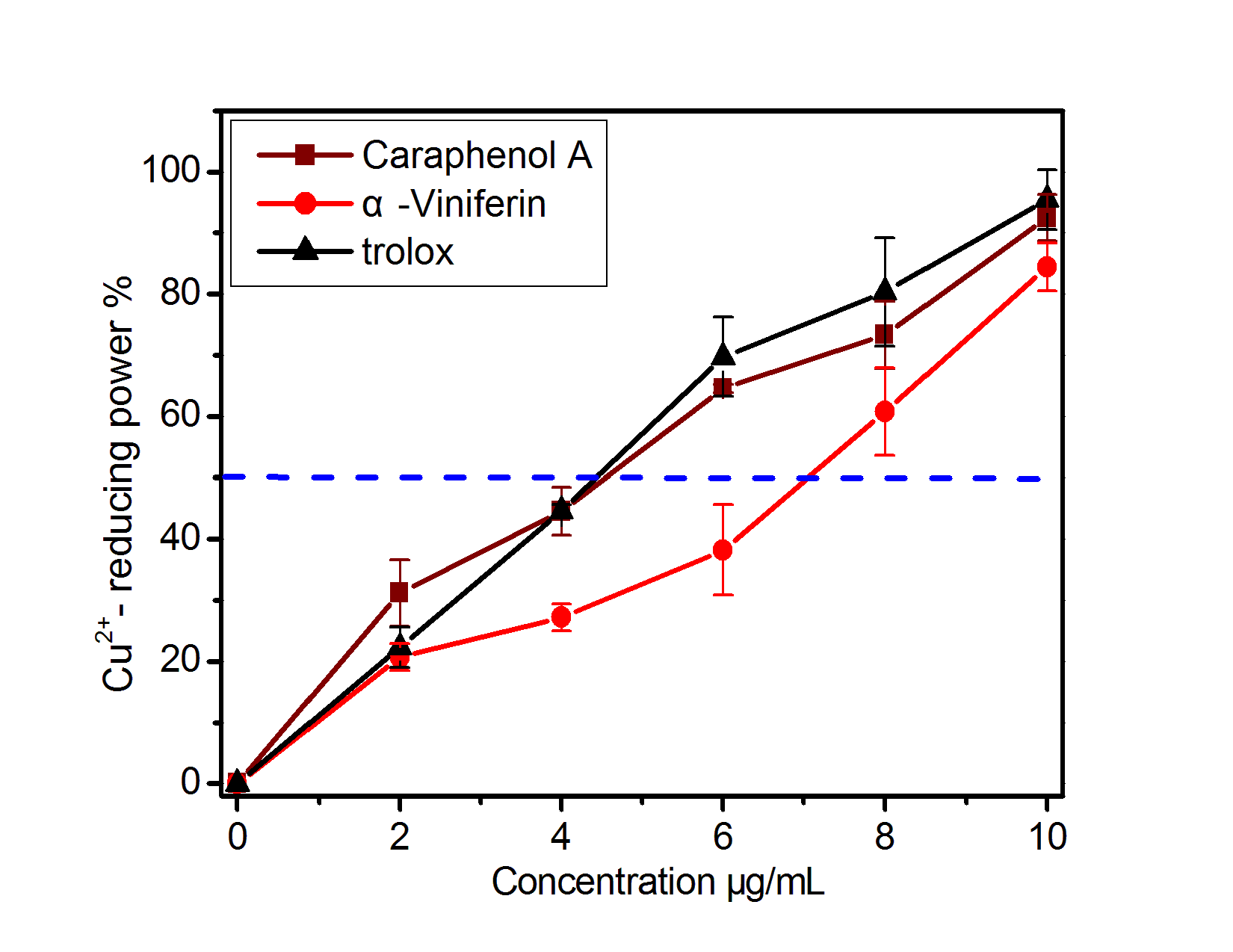


Figure S2**:** The dose response curves of α-viniferin and caraphenol A in Cu^2+^-reducing power assay. Each value is expressed as mean ± SD (n = 3).


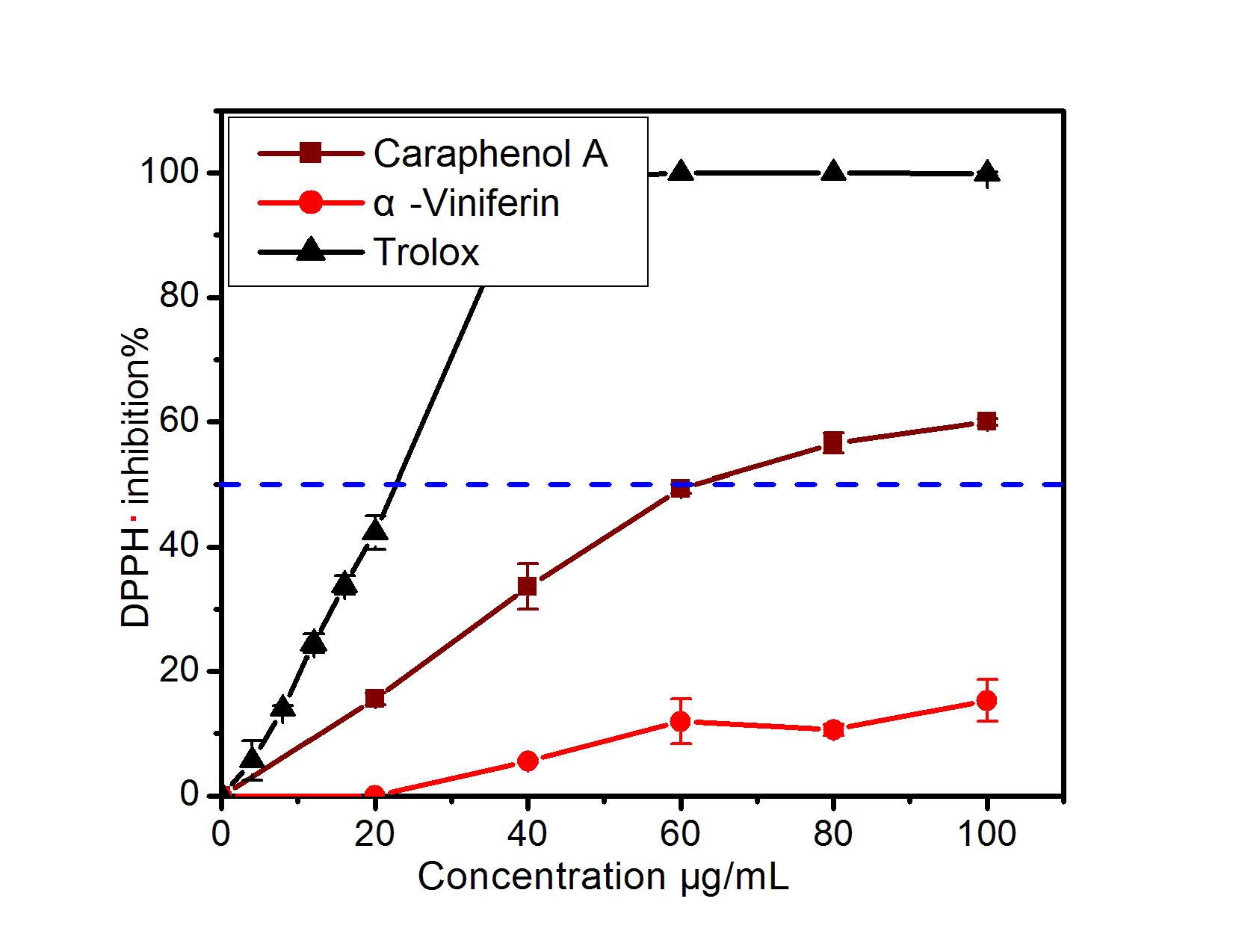


Figure S3**:** The dose response curves of α-viniferin and caraphenol A in DPPH•-scavenging assay. Each value is expressed as mean ± SD (n = 3).


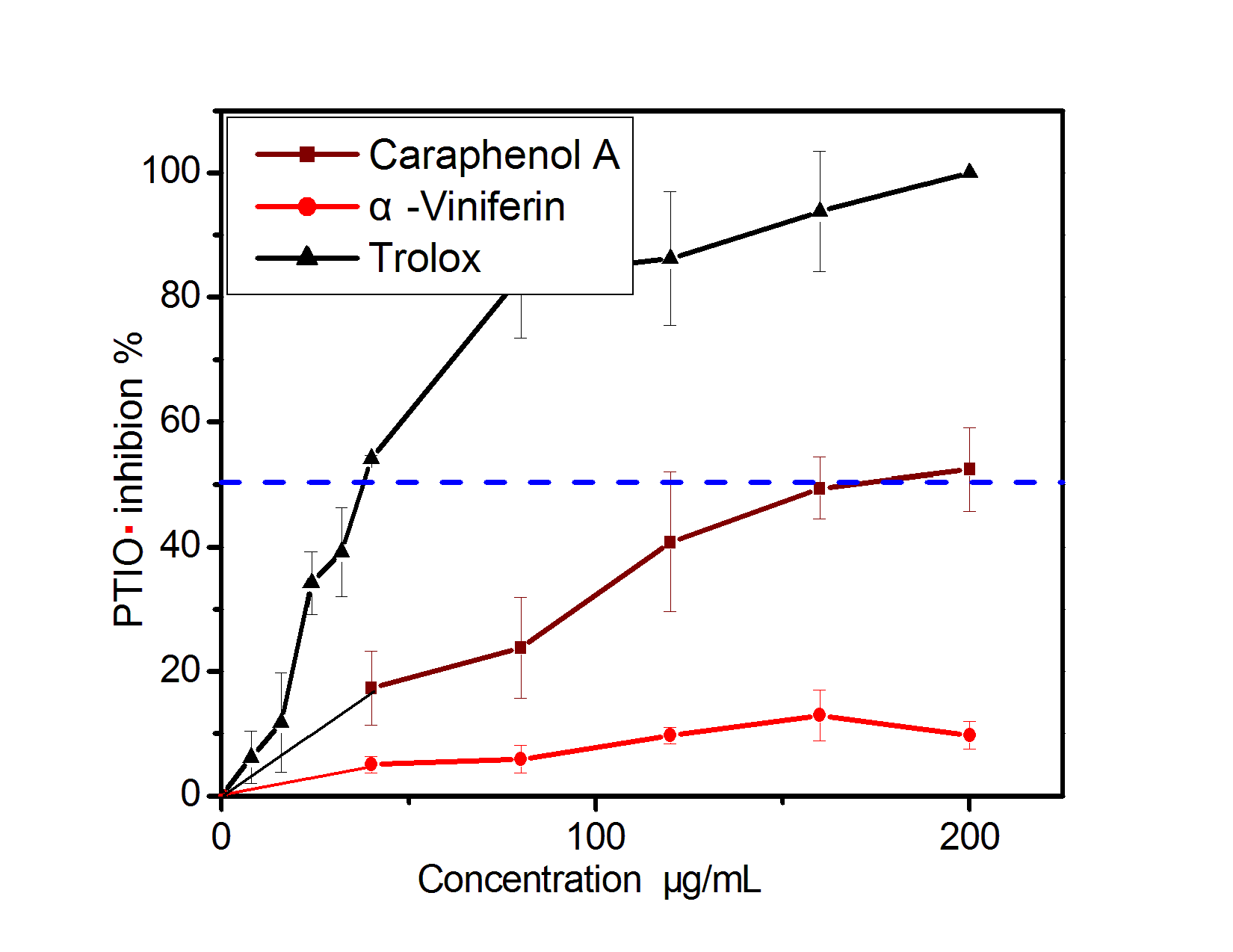


Figure S3**:** The dose response curves of α-viniferin and caraphenol A in PTIO•-scavenging assay. Each value is expressed as mean ± SD (n = 3).
